# CENPA promotes clear cell renal cell carcinoma progression and metastasis via Wnt/β-catenin signaling pathway

**DOI:** 10.1186/s12967-021-03087-8

**Published:** 2021-10-09

**Authors:** Qi Wang, Jiaju Xu, Zhiyong Xiong, Tianbo Xu, Jingchong Liu, Yuenan Liu, Jiaping Chen, Jian Shi, Yi Shou, Changjie Yue, Di Liu, Huageng Liang, Hongmei Yang, Xiong Yang, Xiaoping Zhang

**Affiliations:** 1 Department of Urology, Union Hospital, Tongji Medical College, Huazhong University of Science and Technology, Wuhan, 430022 China; 2 Department of Pathogen Biology, School of Basic Medicine, Tongji Medical College, Huazhong University of Science and Technology, Wuhan, 430030 China; 3 Department of Thoracic, Union Hospital, Tongji Medical College, Huazhong University of Science and Technology, Wuhan, 430022 China

**Keywords:** Kidney renal cell carcinoma, Biomarker, Gene set enrichment analysis, Metastasis, CENPA, Targeted therapy

## Abstract

Clear cell renal cell carcinoma (ccRCC) is the most common malignant tumor of the kidney. New and reliable biomarkers are in urgent need for ccRCC diagnosis and prognosis. The CENP family is overexpressed in many types of cancers, but its functions in ccRCC have not been fully clarified. In this paper, we found that several CENP family members were highly expressed in ccRCC tissues. Also, CENPA expression level was related to clinicopathological grade and prognosis by weighted gene co-expression network analysis (WGCNA). CENPA served as a representative CENP family member as a ccRCC biomarker. Further in vitro experiments verified that overexpression of CENPA promoted ccRCC proliferation and metastasis by accelerating the cell cycle and activating the Wnt/β-catenin signaling pathway. The elevated β-catenin led by CENPA overexpression translocated to nucleus for downstream effect. Functional recovery experiment confirmed that Wnt/β-catenin pathway was essential for ccRCC progression and metastasis. Developing selective drugs targeting CENPA may be a promising direction for cancer treatment.

**Supplementary Information:**

The online version contains supplementary material available at 10.1186/s12967-021-03087-8.

## Background

Renal carcinoma is a malignancy in urinary system with high incidence. As reported by the American Cancer Society’s most recent estimates about renal malignancies in the United States for 2020, approximately 76,080 new cases of kidney and renal pelvis cancer would be diagnosed, and approximately 13,780 people would die from this disease [1]. Renal cell carcinoma (RCC) accounts for 90% of all renal malignancies [2]. Clear cell renal cell cancer (ccRCC) is a major and malignant subtype of renal carcinoma, accounting for approximately 3/4 of RCC [3].

Although ccRCC’s diagnostic technique has been greatly improved, approximately one in three patients have advanced tumor when first diagnosed still have distant metastasis at the time of diagnosis [4]. These patients may have a worse prognosis due to missing the timing for surgery. Beyond surgery, radiotherapy and traditional chemotherapy are not as effective for ccRCC, which is why targeted therapy has been developed. However, insensitiveness and resistance could present problems for the use of traditional molecular targeted antitumor drugs, including sunitinib, a widely applied drug for RCC. Researchers are striving for new targets [5, 6], yet few are sufficiently effective for clinical research. As a result, it is imperative to look for new biomarkers for early diagnosis and targeted therapy.

Sustained proliferative signaling is a distinctive feature of tumors [7]. Mitotic defects accumulation of finally lead to chromosomal instability (CIN) [8]. Cancers are frequently aneuploid [9], and the alteration of oncogenes or tumor suppressors that regulates changes in chromosome number may contribute to tumorigenesis, progression, metastasis, and prognosis of patients [10–12]. Accurate duplication and segregation of our chromosomes depend on precise assemblies of the kinetochore protein complex on centromeric chromatin [13], but abnormal segregation leads to chromosomal instability and aneuploidy [14]. Centromere protein A, namely CENPA or CenH3, is recognized as a marker of centromeric location, as it exists in all active centromeres [15]. Overexpression of CENPA promotes aneuploidy with karyotypic heterogeneity [16]. In contrast, CENPA deficiency drives apoptosis and induces cell cycle arrest [17–20].

Extensive studies have uncovered elevated CENPA levels in tumors and their effect in tumorigenesis, including colorectal cancer [12, 21], breast cancer [22, 23], gastric cancer [24], prostate adenocarcinoma [25], and lung cancer [26]. However, though researchers claimed that CENPA may play a role in kidney cancer through bioinformatics analyses [27–30], the relationship between CENPA and ccRCC has not been unearthed by in vitro experiment yet.

Here we systematically analyzed the role of CENP family members in ccRCC. We found that CENPA, a representative of CENP family member, was highly expressed and could be a diagnostic and prognostic biomarker of ccRCC. In addition, downregulation or upregulation of CENPA could inhibit or promote the proliferation, migration and invasion of ccRCC in vitro. With further exploration, we found that CENPA accelerated cell cycle and activated the Wnt pathway. Finally, functional rescue experiments indicated that CENPA promoted ccRCC cell proliferation and metastasis by activating the Wnt/β-catenin pathway.

## Results

### CENPA was identified as a hub gene in ccRCC via WGCNA

**Fig. 1:**
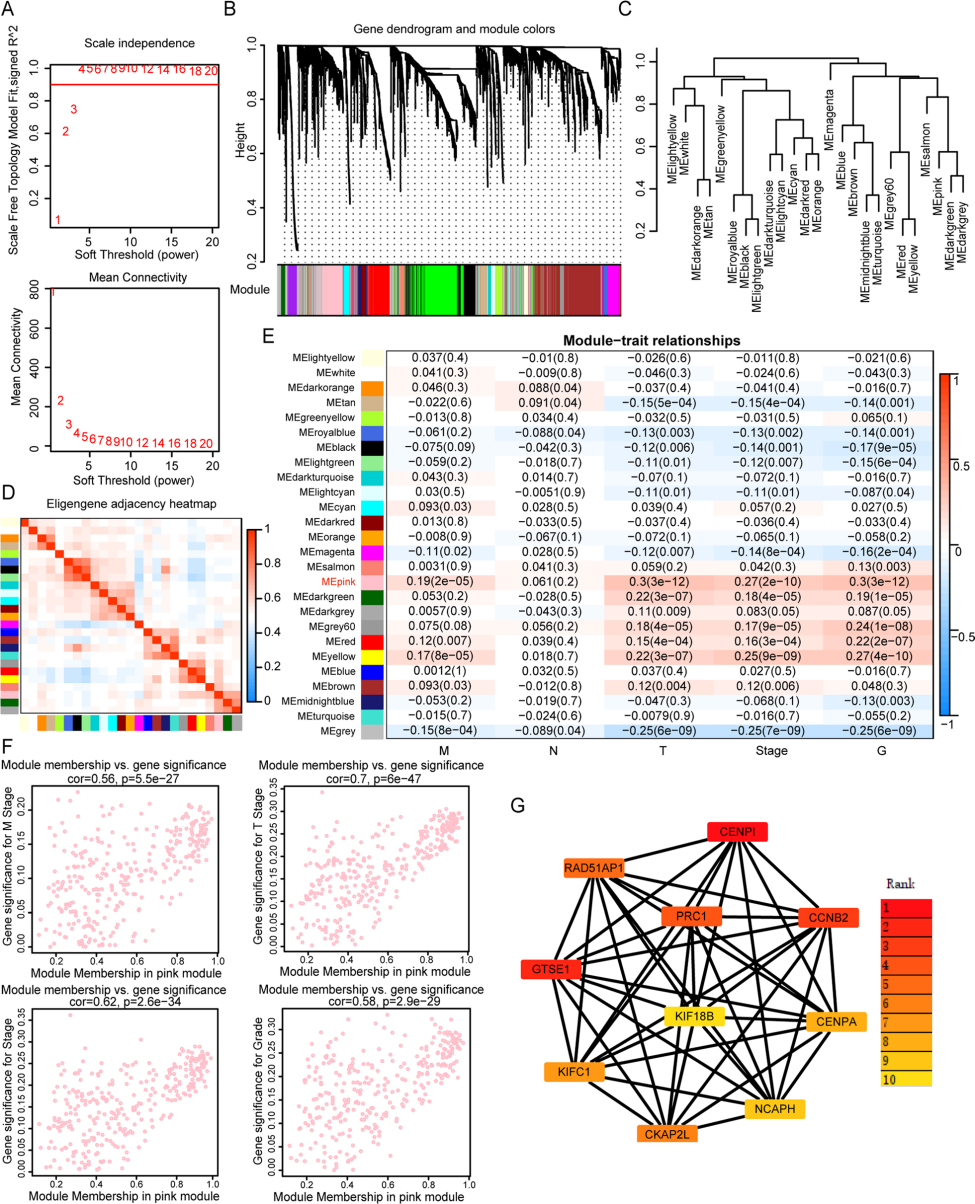
Weighted gene co-expression network analysis (WGCNA) identified hub genes of renal cell carcinoma. **A** Analysis of Scale independence and mean connectivity for various soft-thresholding power. **B** The 26 gene dendrogram and module colors of DEGs based on TOM. **C**, **D** The eigengene adjacency heatmap and clustering of module eigengene to display the relationships between each module. **E** The relationships between 25 modules and clinical traits including T, N, M Stage and G grades. The pink module was selected as the most significant module. **F** The relationships between the genes in pink module and gene significance for M stage, T stage, Stage, and G grade. **G** The network of ten top hub genes by Cytoscape software. DEGs: differentially expressed genes; TOM: topological overlap matrix

**Fig. 2:**
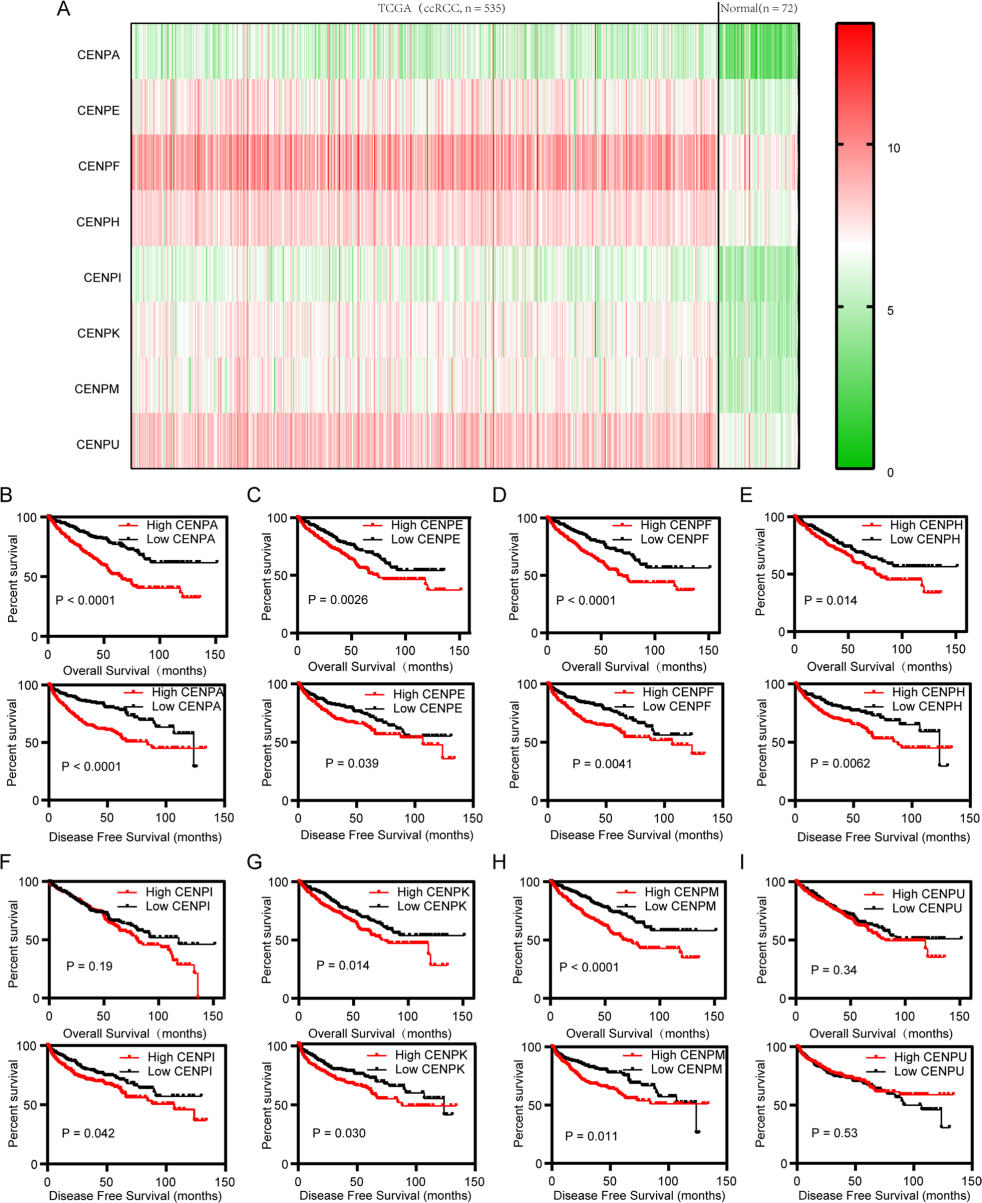
Expression and survival curve of 8 members of CENP family. **A** The expression heatmap of eight members (CENPA, CENPE, CENPF, CENPH, CENPI, CENPK, CENPM, CENPU) of CENP family in TCGA ccRCC. Left: 535 ccRCC tissues; right: 72 cancer-adjacent tissues. **B**–**I** The OS and DFS curve of the eight CENP family members. In each analysis, all patients were sorted in ascending order based on corresponding gene expression, then they were divided into two groups with the same sample size. The OS and DFS of patients in two groups were visualized by Kaplan–Meier plot. OS: overall survival; DFS: disease free survival

To find the hub genes for ccRCC from the analyzed gene set, 6137 genes were identified as differentially expressed genes (DEGs) by the “limma” package according to the cutoff criterion. WGCNA was used to screen hub modules closely related to clinical traits. In our study, a scale-free network was ensured with the soft threshold β = 4 (Fig. 1A). Based on the gene expression pattern, we identified 25 modules shown in Fig. 1B. For correlation coefficient between modules were all less than 0.8 (Fig. 1C, D), no modules needed to be merged. As shown in Fig. 1E, we selected the pink module (T stage: r = 0.3, p = 3e−12; N stage: r = 0.061 p = 0.2; M stage: r = 0.19, p = 2e−5; Stage: r = 0.27, p = 2e−10) as the hub module for further analysis. The relationships between the genes in the pink module for M stage, T stage, N stage, stage, and G grade were presented in Fig. 1F. Notably, the top 10 hub genes included CENPI and CENPA, which are members of the CENP family (Fig. 1G). Then, we focused our interest on the CENP family. Eight members (CENPA, CENPE, CENPF, CENPH, CENPI, CENPK, CENPM, CENPU) of the CENP family were all upregulated in KIRC (kidney renal clear cell carcinoma) cohort from TCGA (The Cancer Genome Atlas) database (Fig. 2A). The plot of genetic alteration suggests that genes in CENP family seldomly mutate (Additional file 2: Figure S2I). From the Kaplan–Meier curve for overall survival (OS) and the disease-free survival (DFS) of the eight genes in TCGA-KIRC, we found that neither CENPI nor CENPU had prognostic significance (Fig. 2B–I). Although the remaining 5 genes (CENPE, CENPAF, CENPH, CENPK, and CENPM) had prognostic value, they were not hub genes by WGCNA analyses. Then subgroup analysis according to age, gender, Stage and G grade were consistent with the previous (Additional file 1: Fig. S1D–G). Above all, CENPA was not only a hub gene, but could also predict the prognosis of ccRCC patients, indicating it to be a representative gene of the CENP family in ccRCC.

### The expression level of CENPA was significantly associated with clinicopathological features

**Fig. 3:**
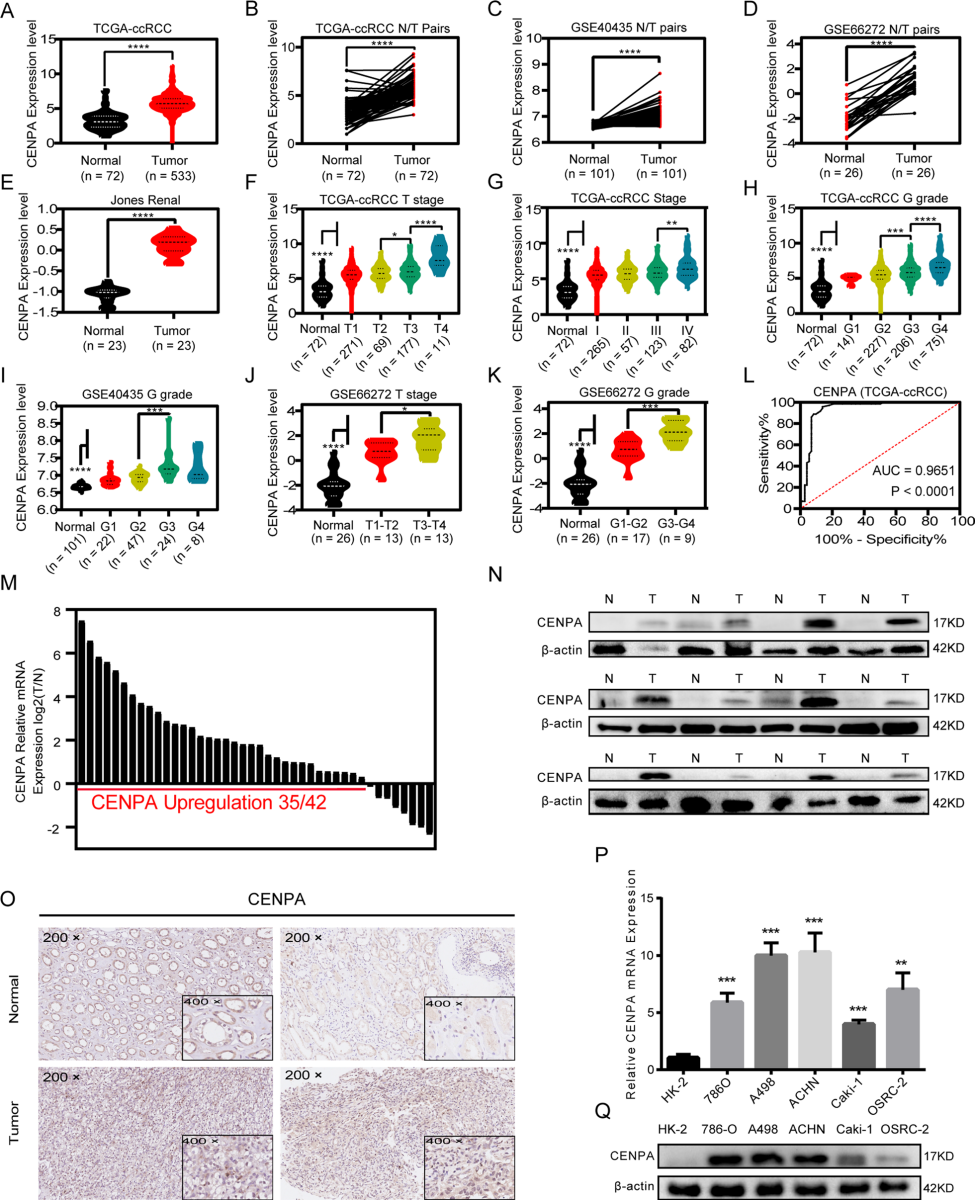
CENPA was closely related to clinical traits and overexpressed in ccRCC tissues and cells. Public datasets showed CENPA overexpression in ccRCC compared to normal tissues. **A** In TCGA-KIRC cohort, 533 ccRCC samples showed higher CENPA expression than 72 normal samples. For tissues gathered from the same patients, CENPA overexpressed in cancer tissues compared to cancer-adjacent tissues in **B** TCGA-KIRC dataset (72 pairs of samples), **C** GSE40435 dataset (101 pairs of samples), **D** GSE66272 dataset (26 pairs of samples) and **E** Jones Renal dataset (23 pairs of samples). The expression of CENPA elevated with various clinicopathological factors in public datasets, including **F**, **J** T stage (528 samples in TCGA-KIRC and 26 samples in GSE66272), **G** AJCC clinical stage (527 samples in TCGA-KIRC), and **H**, **I**, **K** G grade (522 samples in TCGA-KIRC, 101 samples in GSE40435 and 26 samples in GSE66272). **L** The ROC curve of CENPA expression (AUC = 0.9651; p < 0.0001) in TCGA-KIRC cohort. Our own cohort validated CENPA overexpression in ccRCC through **M** qRT-PCR assays (42 pairs), **N** immunoblotting tests (12 pairs), and **O** immunohistochemical analyses (2 pairs). CENPA overexpressed in renal cancer cell lines (786-O, A498, ACHN, Caki-1 and OSRC-2) compared to normal renal cell line (HK-2) via **P** qRT-PCR and **Q** immunoblotting tests. Relative *p < 0.05; **p < 0.01; ***p < 0.001; ****p < 0.0001. Error bars indicate mean ± SD. AUC: areas under the curve

Next, we aimed to investigate the aberrant expression of CENPA in ccRCC. Firstly, we investigated the mutations and copy-number alterations (CNAs) of CENPA. As illustrated above, there exist no genetic alterations for CENPA in TCGA-KIRC cohort (Additional file 2: Figure S2I). We then explored the CCLE (Cancer Cell Line Encyclopedia), finding that no mutations or CNAs were detected in kidney cancer cell lines. Transcriptomically, CENPA was overexpressed in tumor tissues in the TCGA-KIRC project (Fig. 3A-B), GEO (gene expression omnibus) database (Fig. 3C, D) and Oncomine database (Fig. 3E) [31–33]. Also, CENPA overexpression is not acquired from treatment as we can see a similar result when we eliminated samples from patients with adjuvant therapy prior to the surgery (Additional file 1: Figure S1L-M). As shown in Fig. 3F–H and Additional file 1: Figure S1A-B, the expression of CENPA was positively correlated with multiple clinical stages (T stage, N stage, M stage and TNM stage and G stage). Similar results were obtained in other datasets (Fig. 3I–K). Higher CENPA expression indicated shorter survival time and higher tumor grade and stage (Table 1). Univariate and multivariate analyses were conducted showing that CENPA was one of the independent prognostic markers of ccRCC (Table 2). In addition, ROC (receiver operator characteristic) curve analysis showed that CENPA could be used as a good diagnostic marker (Fig. 3L and Additional file 1: Figure S1C). Furthermore, the ROC curve analyses were conducted between clinicopathological subgroups such T1+2 vs T3+4, M0 vs M1, Stage I+II vs Stage III+IV, and G1+2 vs G3+4 (Additional file 1: Figure S1H–K), which indicated good diagnostic value of CENPA expression for clinicopathological subgroups. Thus, CENPA can serve as a potential diagnostic and prognostic biomarker in ccRCC.

**Table 1:** Correlation between CENPA mRNA expression and clinicopathological parameters of ccRCC patients

Parameter	Number	CENPA mRNA expression		P-value
		Low (n = 261)	High (n = 261)	
Age (years)				
< 60	235	120	115	0.725
≥ 60	287	141	146	
Sex				
Female	183	108	75	0.0033
Male	339	153	186	
T stage				
T1 or T2	336	197	139	< 0.0001
T3 or T4	186	64	122	
N Stage				
N0 or Nx	507	258	249	0.0326
N1	15	3	12	
M Stage				
M0 or Mx	445	242	203	< 0.0001
M1	77	19	58	
G grade				
G1 or G2 or Gx	245	157	88	< 0.0001
G3 or G4	277	104	173	
TNM stage				
I + II	318	190	128	< 0.0001
III + IV	204	71	133	

The four-grid tables were made according to clinicopathological characteristics and the CENPA expression level. Statistical analyses were conducted via Pearson's χ2 test. P < 0.05 was considered statistically significant

**Table 2:** Univariate and multivariate Cox regression analyses of CENPA mRNA level and patient overall survival (OS)

Variable	Univariate analysis			Multivariate analysis^c^		
	HR^a^	95% CI^b^	P	HR	95% CI	*P*
Overall survival (n = 522)						
CENPA						
Low (n = 261)	2.384	1.722–3.3	0.003	1.656	1.17–2.344	0.004
High (n = 261)						
Age						
< 60 (n = 235)	1.641	1.196–2.252	0.002	1.391	1.003–1.928	0.048
≥ 60 (n = 287)						
Gender						
Female (n = 183)	1.071	0.783–1.464	0.669	1.239	0.894–1.717	0.198
Male (n = 339)						
T stage						
T1 or T2 (n = 336)	3.184	2.336–4.329	< 0.001	1.626	1.127–2.347	0.009
T3 or T4 (n = 186)						
N stage						
N0 or NX (n = 507)	3.96	2.143–7.315	< 0.001	2.087	1.101–3.956	0.024
N1 (n = 15)						
M stage						
M0 or MX (n = 445)	4.378	3.199–5.992	< 0.001	2.52	1.752–3.625	< 0.001
M1 (n = 77)						
G grade						
Gx or G1 or G2 (n = 245)	2.681	1.901–3.782	< 0.001	1.638	1.129–2.377	0.009
G3 or G4 (n = 277)						

^a^Hazard ratio, estimated from Cox proportional hazard regression model

^b^Confidence interval of the estimated HR

^c^Multivariate models were adjusted for T, N, M stage, G grade, age and gender

### CENPA was upregulated in ccRCC tissues and cells

To verify the expression levels of CENPA in ccRCC tissues, qRT-PCR (reverse transcription-quantitative polymerase chain reaction) and IBT (immunoblotting test) were performed. It was observed that CENPA expression levels were notably elevated in tumor tissues in comparison with their corresponding adjacent normal tissues (Fig. 3M, N). The immunohistochemistry (IHC) results of cancer/para-cancer pairs also suggested that CENPA was upregulated in cancer tissues (Fig. 3O). Furthermore, we confirmed that the mRNA and protein levels of CENPA were higher in RCC cell lines (786-O, A498, ACHN, Caki-1 and OSRC-2) than in the normal renal cell line HK-2 by qRT-PCR and IBT (Fig. 3P, Q). Generally, these results collectively pinpoint the fact that CENPA is overexpressed in ccRCC.

### CENPA promoted the proliferation, invasion and migration of ccRCC cells in vitro

**Fig. 4:**
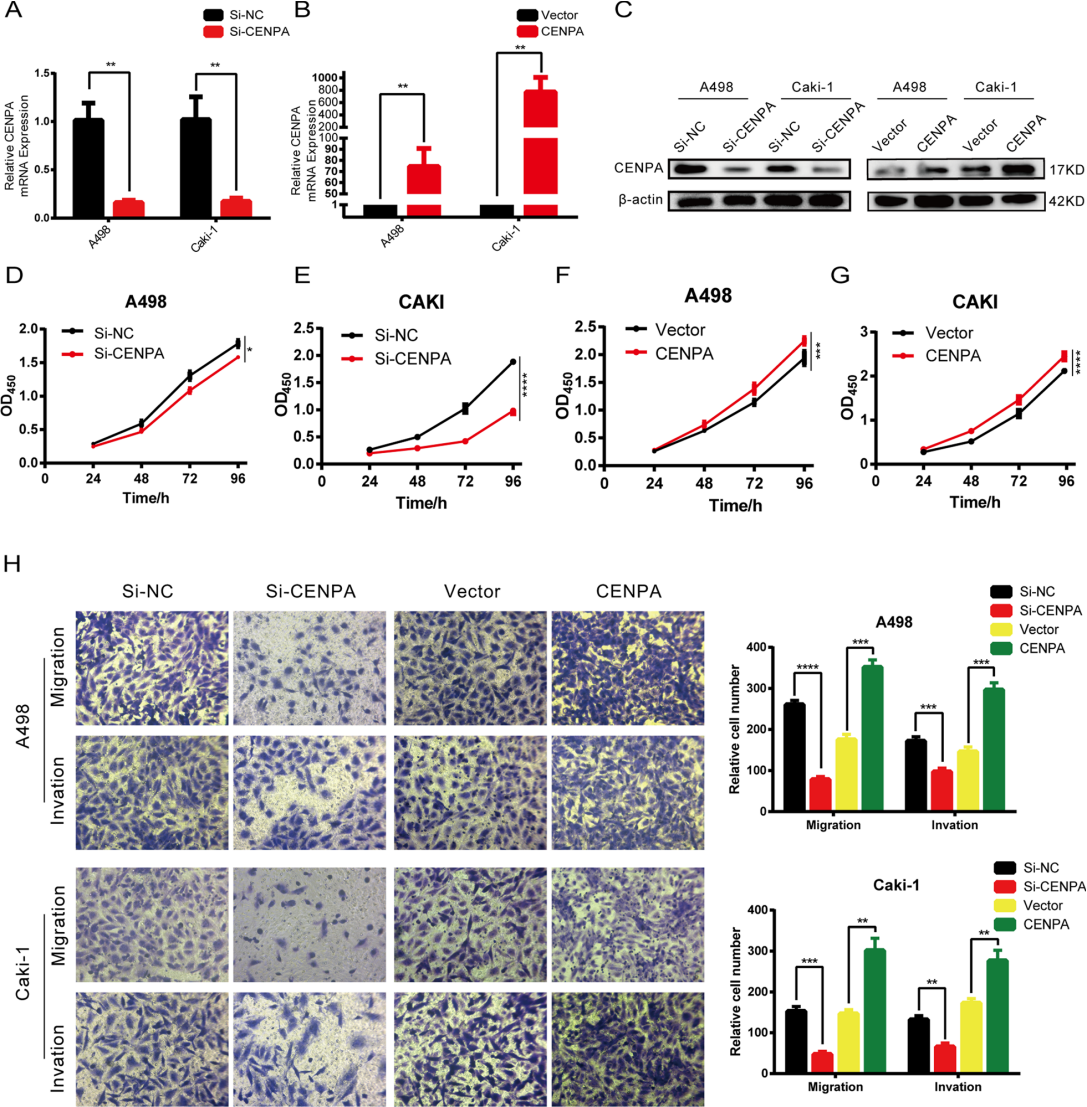
CENPA promoted the proliferation, migration and invasion of ccRCC cells. CENPA **A**, **B** mRNA (n = 3 per group) and **C** protein levels were verified by qRT-PCR in A498 and Caki-1 cells with transient CENPA knockdown or overexpression. **D**–**F** Cell viability of A498 and Caki-1 cells after depleting or overexpressing CENPA was calculated using CCK-8 assays for four days (n = 6 per group). **H** The cell migration and invasion ability of transfected A498 and Caki-1 cells were evaluated using transwell assays (Magnification: 200×, n = 3 per group). *p < 0.05; **p < 0.01; ***p < 0.001; ****p < 0.0001. Error bars indicate mean ± SD

**Fig. 5:**
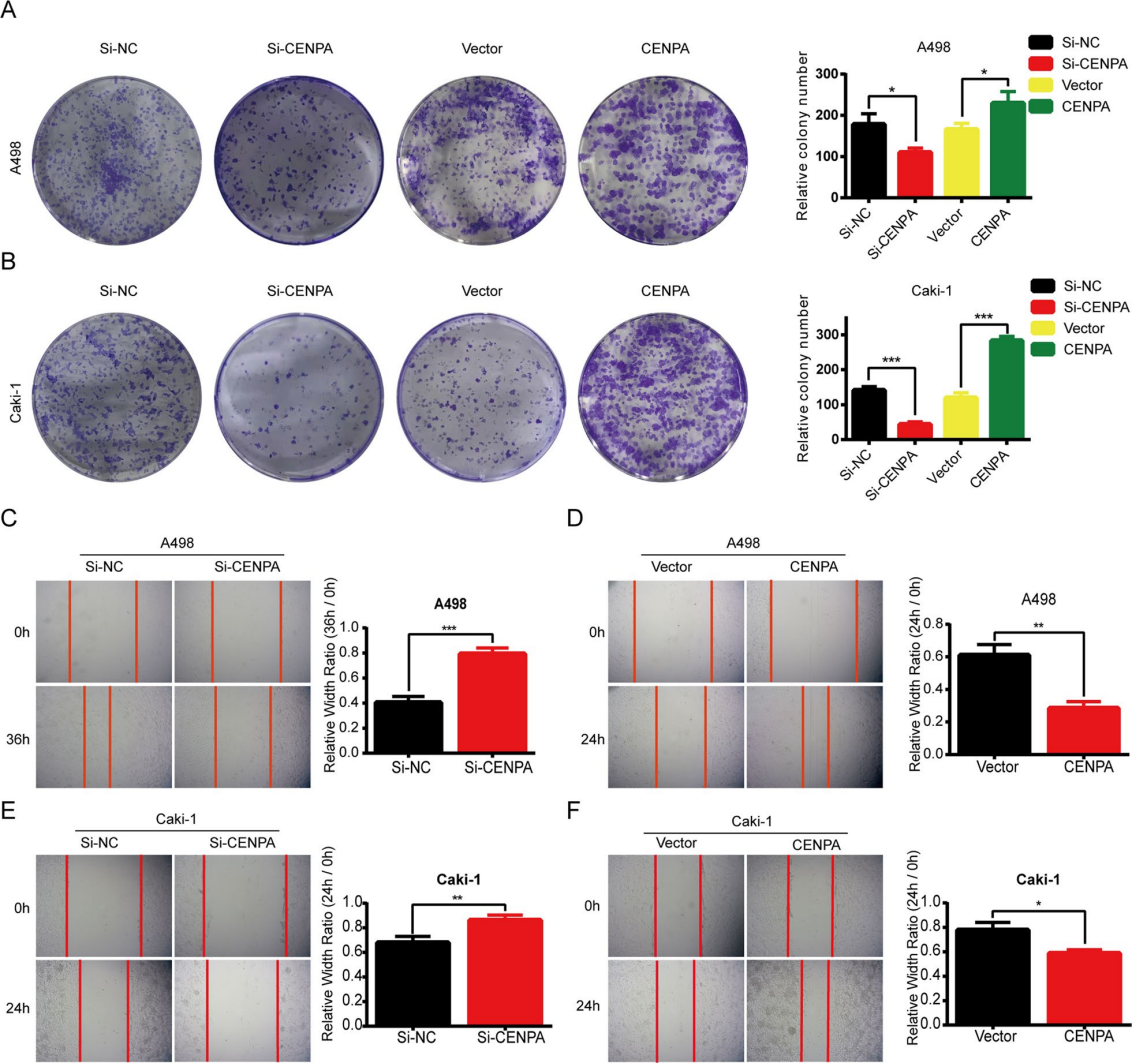
CENPA promoted the proliferation and migration of ccRCC cells. **A**, **B** The colony formation ability of transfected A498 and Caki-1 cells was evaluated by colony numbers for 12 days after seeding 1000 cells in the culture dish. The assays were independently conducted in triplicate. **C**–**F** The migration ability of transfected ccRCC cells was evaluated by wound healing assays. The cells were wounded by a 10-μl pipet when reaching 100% confluence. The images were taken 24 h or 36 h later. *p < 0.05; **p < 0.01; ***p < 0.001; ****p < 0.0001. Error bars indicate mean ± SD

To investigate the effect of CENPA on the biological behaviors of ccRCC, ccRCC cell lines were transfected with si-CENPA or CENPA plasmid to down- or upregulate the expression of CENPA. The mRNA and protein expression levels decreased or increased significantly in A498 and Caki-1 cells compared with the corresponding negative control (Fig. 4A–C). CCK-8 (cell counting kit-8) assays suggested that tumorous cells downregulated or upregulated in CENPA inhibited or promoted proliferation, respectively (Fig. 4D–G). The colony formation assays confirmed this finding (Fig. 5A, B). In addition, transwell assays (Fig. 4H) and wound healing assays (Fig. 5C–F) collectively indicated that the level of CENPA was positively correlated with the migration and invasive abilities of the cells. These results provided us with solid evidence that CENPA promoted the proliferation, migration and invasion of ccRCC cells, which is significant in the cascade of tumor metastasis.

### CENPA activated the Wnt/β-catenin pathway and accelerated the cell cycle

**Fig. 6:**
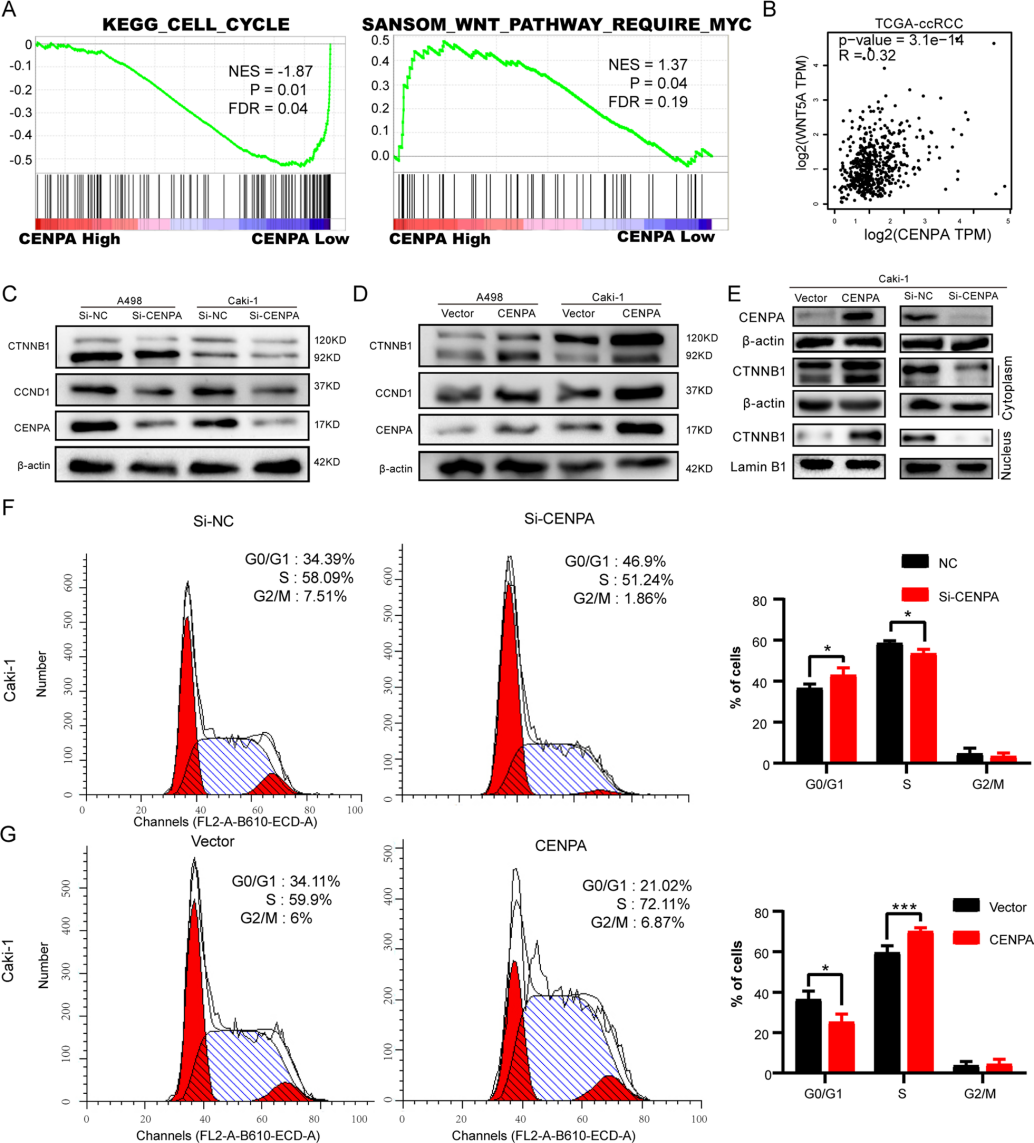
CENPA accelerated the cell cycle and activated the Wnt signaling pathway. **A** The GSEA results of CENPA using TCGA-KIRC expression dataset. 533 tumoral samples was divided into two groups based on CENPA level. Genes expression patterns of two groups was different in cell cycles and Wnt pathway. **B** The expression of WNT5A is positively correlated to the expression of CENPA in TCGA-KIRC ccRCC tissues. **C**, **D** The expression of cyclin D1(CCND1) and β-catenin (CTNNB1) were down-regulated or upregulated in ccRCC cells with knockdown or overexpression of CENPA. **E** Western blot revealed the expression of CTNNB1 in the cytoplasm and nucleus. β-actin and Lamin B1 were used as internal references in the cytoplasm and nucleus, respectively. **F**–**G** Cell cycle distribution was analyzed by PI staining in Caki-1 cells after transfection by Si-CENPA or CENPA plasmid for 48 h. *p < 0.05; **p < 0.01; ***p < 0.001; ****p < 0.0001. Error bars indicate mean ± SD

To determine how CENPA is involved in ccRCC pathogenesis, functional enrichment analyses were performed using the TCGA-KIRC cohort to identify ccRCC-related pathways and biochemical processes affected by differentially expressed CENPA. GSEA (Gene Set Enrichment Analysis) results indicated that the high expression of the CENPA group was mainly enriched in cell cycle pathways and Wnt pathways (Fig. 6A). The results of GO (Gene Ontology) and KEGG (Kyoto Encyclopedia of Genes and Genomes) analyses also included cell cycle pathways (Additional file 2: Figure S2A, B). We found that the expression of CENPA was positively related to WNT5A using GEPIA in TCGA-KIRC, which encodes a member of the Wnt family that signals through both the canonical and noncanonical Wnt pathways (Fig. 6B). To verify the presumption of bioinformatics analysis, the western blotting assays were performed, and we found that the silencing of CENPA could significantly downregulate the expression of β-catenin (CTNNB1) and its target gene cyclin D1 (CCND1) in ccRCC (Fig. 6C), whereas the overexpression of CENPA could upregulate the expression of β-catenin (CTNNB1) and cyclin D1 (CCND1) in ccRCC (Fig. 6D). Then, the β-catenin (CTNNB1) nuclear accumulation was observed with CENPA overexpression whereas CENPA knockdown reduced nuclear proportion of β-catenin (CTNNB1) (Fig. 6E). In addition, cell cycle assays showed that Caki-1 cells were accumulated in G0/G1 phase, suggesting an inhibition of cell cycle from G0/G1 to S phase (Fig. 6F). The cell cycle arrest blocked cell growth. In contrast, overexpression of CENPA promoted entry of more cells into S phase so that the cell proliferation rate increased (Fig. 6G).

### The Wnt/β-catenin pathway is involved in CENPA-mediated proliferation and metastasis

**Fig. 7:**
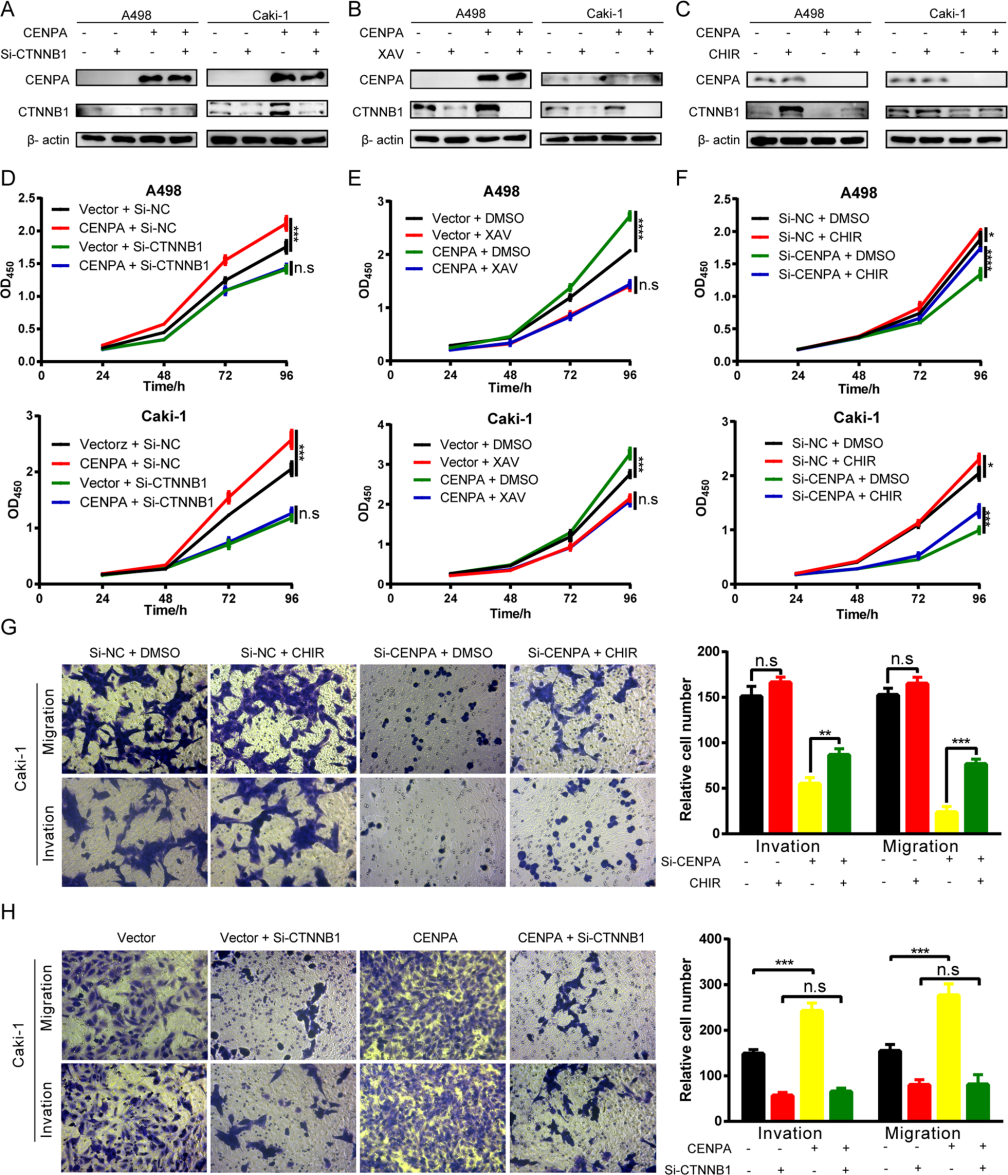
The Wnt/β-catenin pathway is involved in CENPA-mediated proliferation and metastasis. **A** A498 and Caki-1 cells with CENPA overexpression were treated with Si-CTNNB1 for 48 h. Then, expression of CTNNB1 was measured by western blotting. **B**, **C** A498 and Caki-1 cells with CENPA overexpression or knockdown were treated with Wnt-pathway inhibitor XAV-939 (10 μmol/L) or agonist CHIR-99012 (10 μmol/L) for 24 h. Then, western blotting determined that the drugs were effective to Wnt pathway. **D**–**F** Cell viability was assessed in A498 and Caki-1 cells with CENPA overexpression and Wnt pathway inhibition/excitation via CCK-8 assays (n = 4 per group). **G**, **H** Caki-1 cells with overexpression or knockdown of CENPA were treated with Si-CTNNB1 for 48 h or CHIR-99021 (10 μmol/L) for 24 h as indicated and subjected to migration assay and invasion assay (magnification: 200×, n = 3 per group). NC: negative control; n.s: not significant; *p < 0.05; **p < 0.01; ***p < 0.001; ****p < 0.0001. Error bars indicate mean ± SD

To further test whether the Wnt/β-catenin pathway was required for the downstream effect of CENPA on cell proliferation and metastasis, we conducted in vitro rescue experiments. We used Si-CTNNB1 to knock down the expression of β-catenin (CTNNB1) (Additional file 2: Figure S2D, G). And the Wnt/β-catenin inhibitor XAV-939 or activator CHIR-99021 trihydrochloride were utilized to inhibit or activate the Wnt/β-catenin pathway respectively. To explore their effects on β-catenin (CTNNB1), a series of concentration gradients for 24 h and time gradients for 10 μM of XAV-939 and CHIR-99021 trihydrochloride were employed Caki-1 (Additional file 2: Figures S2C, E, F, H). Then, 10 uM and 24 h were considered to be a proper drug treatment condition. Following depletion of β-catenin by transfection of siRNAs, the function of CENPA on cell proliferation was reduced, as shown by CCK-8 assays (Fig. 7A, D). A similar result was also obtained by using XAV-939 (Fig. 7B, E). Moreover, following activation of Wnt/β-catenin signaling with the CHIR-99021 trihydrochloride, the proliferative ability of CENPA-depleted cells was enhanced (Fig. 7C, F). Therefore, we believe that CENPA promotes the progression of ccRCC through activating the Wnt/β-catenin signaling pathway. In addition, to investigate whether CENPA exerted its effects during ccRCC metastasis in the context of the Wnt/β-catenin pathway, we conducted transwell assays. Similar to CCK-8 assays, inhibition of β-catenin reduced CENPA-mediated migration and invasion (Fig. 7G). In contrast, activation of the Wnt/β-catenin pathway enhanced the abilities in CENPA-depleted cells (Fig. 7H). These results indicated that CENPA promoted ccRCC cell proliferation and metastasis by activating the Wnt/β-catenin pathway.

## Discussion

Here, we uncovered CENPA as a new ccRCC biomarker and demonstrated that CENPA acts crucially in ccRCC. First, through performing WGCNA analyses, we found that eight CENP family members (CENPA, CENPE, CENPF, CENPH, CENPI, CENPK, CENPM, CENPU) were closely related to clinical stage and grade with a similar expression pattern in pink module. Second, we selected the CENPA as a representative of the eight CENP family members. Functional experiments proved that CENPA could accelerate RCC cell proliferation and metastasis. Additionally, our experimental evidence showed that upregulating CENPA could accelerate the cell cycle and trigger the Wnt/β-catenin pathway. Finally, functional rescue experiments indicated that CENPA promoted ccRCC cell multiplication and metastasis through triggering the Wnt/β-catenin pathway.

CENPA exert a key role during mitosis, which epigenetically determines the position of sister chromatids by determining the position of the centromere on chromosome epigenetically [18, 34]. CENPA overexpression leads to mislocalization in noncentromeric regions, resulting in chromosome segregation, aberrations and genome instability [13, 35]. Interestingly, High-level CENPA in ccRCC is consistent with the CIN in cancer. In addition, CENPA overexpression promotes aneuploidy with karyotypic heterogeneity [36]. Therefore, we deduced that chromosomal aneuploidy caused by CENPA overexpression is an important cause of ccRCC.

Due to the limitations of current treatment, researchers have focused on targeted therapy, and research on ccRCC pathogenesis and the search for new therapeutic targets are increasing; nonetheless, few of them achieve clinical usage. Thus, we aimed to uncover a new mechanism of ccRCC progression and discuss its potential application for developing new drugs. According to our results, CENPA acts crucially in ccRCC genesis and progression, so it maybe a potential target. Presently, some antitumor drugs that target key molecules in cell division have been developed. PF-2771 [37] and GSK923235 [38] are both CENPE inhibitors. Ispinesib could specifically inhibit kinesin spindle protein [39]. But they are all investigational. Due to CENPA overexpression in several types of cancers, it is expected to be a broad-spectrum anti-tumor target in clinical use. Research on anti-cancer drugs target Wnt/β-catenin pathway has been advancing. Schultz-Hausmann et al. confirmed that ethacrynic acid, ciclopirox olamine and piroctone olamine had cytotoxic effect on RCC cell lines via Wnt/β-catenin pathway [40], but they are not specific inhibitors of the pathway. The side effects of them are unable to predict. Several researchers have developed more specific drugs target Wnt/β-catenin pathway such as MSAB [41] and CWP232228 [42], yet they are still far from the patients. New drug research and development target CENPA and Wnt/β-catenin pathway will be a follow-up issue worthy of attention.

Histone variants are considered critical in malignant transformation in several cancer types. As one of the histone H3 variants, CENPA acts crucially in mitosis and contributes to tumor occurrence and development [13, 21, 25, 43]. Past research has mainly focused on the changes and functions during mitosis [44]. With regard to diseases or cancer, it is currently thought that CENPA functions downstream of the pathway or axis rather than upstream [45]. Jeffery et al. uncovered that CENPA overexpression impacted epidermal-mesenchymal transition or radiosensitivity depends on p53 status in cervical or colorectal cancer cell lines [46]. Some studies have shown that histone variants, including CENPA, can act as transcription factors [25, 47]. In kidney cancer, many scholars have already screened out CENPA as a diagnostic and prognostic biomarker through bioinformatics analysis, including chromophobe [27] and ccRCC [28–30]. However, how CENPA functions in ccRCC has not been completely determined. In this study, one of our major contributions is our discovery that artificially regulating the expression of CENPA can not only affected the proliferation and metastasis in ccRCC but change the activity of the Wnt signaling pathway.

The Wnt pathway is involved in many biological processes, including cell differentiation, proliferation, migration, and cell adhesion. Dysregulation of Wnt signal transduction is suggested to be related to various human cancers, including RCC [48]. Many Wnt members were identified as biomarkers for RCC, and some of them were verified as participants in RCC development [49–52]. Piotrowska et al. compared the activation of Wnt/β-catenin pathway among ccRCC, papillary RCC and chromophobe RCC via immunohistochemistry, finding that the Wnt pathway pronouncedly activated in ccRCC [53]. A classical mechanism of the Wnt pathway is to decrease the amount of phosphorylated GSK3β and cytoplasmic β-catenin as well as upregulate many transcription factors that could upregulate oncogene MYC and CCND1 [54]. The Wnt pathway abnormally activates during RCC genesis, and inhibition of the pathway can reduce invasion, migration and drug resistance [55, 56]. In this study, we determined that a high level of CENPA promoted the multiplication and metastasis of RCC by activating the Wnt pathway. CENPA overexpression up-regulates β-catenin, promoting its accumulation in the nucleus and transactivating Cyclin D1. The possible Wnt subunit that CENPA activates may be WNT5A. Functional recovery experiment confirmed that Wnt/β-catenin pathway was essential for ccRCC progression and metastasis.

Due to the important role CENPA plays in cell division, we can easily associate it with cell cycle regulation. According to our results, we uncovered CENPA could up-regulate CCND1, which is a downstream target gene of Wnt pathway. The CCND1 is always overexpressed in cancer [57, 58] and regulates cell cycle transition from G1 phase to S phase along with CCND2 and CCND3 [59]. Consistent with the results above, we found CENPA could progresses cell cycle from G0/G1 phase to S phase that mediated by CCND1.

Our study has some limitations. We only verified the tumor-promoting effect of CENPA through in silico and in vitro experiments without in vivo data. Regarding the mechanisms, we did not determine how Wnt pathway and cell cycle-related proteins regulated by CENPA. The above issues will be the focus of our further research.

## Conclusion

In conclusion, our study unearthed that high-level CENP family genes were related to adverse survival and high clinicopathological stage in ccRCC patients as determined by WGCNA analyses. High-level CENPA could increase the multiplication, migration and invasion ability of ccRCC cells via activating Wnt/β-catenin pathway in vitro. Our effort disclosed that CENPA is an important renal cancer biomarker and a possible highly specific therapeutic target.

## Materials and methods

### Dataset

The data we analysed were obtained from TCGA project (https://portal.gdc.cancer.gov/), UCSC Xena browser (https://xenabrowser.net/), GEO database (GSE44035, GSE66272; https://www.ncbi.nlm.nih.gov/geo/), Oncomine database (Jones Renal dataset; https://www.oncomine.org), and CCLE, which included mutations and CNA data, gene expression datasets (RNA sequencing, RNA-seq), corresponding clinicopathological information and survival (including DFS and OS) information of KIRC patients [60].

### WGCNA

DEGs was acquired by “limma” package [61] under the condition of p < 0.05and |log FC| (|log Fold Change|) > 1.0. The “WGCNA” package was used to construct the co-expression network in R [62]. WGCNA analysis was conducted based on the previously described standard method [6]. All of the above were performed by R 4.0.2. The ten top hub genes were identified by Betweenness method in CytoHubba plugin using Cytoscape software [63].

### Survival and ROC curve analysis

The ccRCC samples were classified into two groups with the same sample size based on the median CENPA mRNA level. DFS and OS were visualized by Kaplan–Meier plot using GraphPad 8.01. Meanwhile, ROC curves were also drawn among two groups. Then, p < 0.05 was considered statistically significant.

### ccRCC tissue samples

120 pairs of ccRCC and their tumor-adjacent renal tissues were acquired from patients at the Department of Urology, Union Hospital in Wuhan during 2015 and 2018. All patients did not undergo adjuvant therapy before the surgery. The clinicopathological features of the 120 ccRCC patients were collected in Additional file 3: Table S1. Our study comply with the regulations of the Human Research Ethics Committee of Huazhong University of Science and Technology. All the procedures in our research obeyed the Declaration of Helsinki. The tumor-adjacent normal renal tissues were taken more than 2.5 cm away from the cancer tissue. The RNAs from 42 paired samples were analyzed by qRT-PCR and proteins extracted from 12 pairs were analyzed via immunoblotting test. Three pairs of samples were analyzed via IHC.

### Cell culture

The human normal cell line HK-2, and five types of ccRCC cell lines (786-O, ACHN, A498, Caki-1 and OSRC-2) got from the institution named American Type Culture Collection were employed in the research. A kind of commonly used culture medium, high glucose Dulbecco's Modified Eagle's Medium (Gibco, USA) was used to culture the cells. Before use, we add 10% fetal bovine serum (Gibco in USA) to the medium. Usually, cells were incubated with 5% CO_2_ at 37.3 °C.

### Immunoblotting test (IBT)

Tissues and cells were lysed in RIPA Buffer (Beyotime, China) including protease inhibitor PMSF (Servicebio, China) for 30 min. The nucleoproteins and plasma proteins were extracted by PARIS™ Kit Protein and RNA Isolation System (Invitrogen, Carlsbad, Canada) directed by the manufacturer’s protocols. Then, BCA Protein Assay Kit (Beyotime in China) was applied for protein quantification. In IBT assays, the condition of 12% gel (SDS-PAGE) at 90 V for 30 min and 120 V for 50 min was used for electrophoresis and the condition of 250 mA for 50 or 90 min was employed for transferring to membrane. After blocked with 5% BSA for 1–2 h at 20 ℃, the membrane was incubated with specific CENPA primary antibody (1:2000; Abclonal in China, A15995), beta-actin (1:5000; Proteintech in China, 66009-1-lg), CCND1(1:2000; Abclonal in China, A19038), CTNNB1(1:2000; Abclonal in China, A11512) and LaminB1(1:1000; Abclonal in China, A16909)at 4 °C for 12 h. After washed with 0.1% PBST (phosphate buffered saline tween) for 10–15 min thrice, the membranes were immersed with specie-matched secondary antibodies (1:2500; Abclonal, China, AS014 and AS003) for 2 h at 25 °C. Finally, following washed with 0.1% PBST for 30–40 min, the bands for each protein were showed with Electrochemiluminescence IBT Substrate (Ultra sensitivity; Biosharp, China) via ChemiDoc-XRS + (Bio-Rad, China). All the original western blot pictures were included in Additional file 4

### RNA extraction and qRT-PCR

Directed by the manufacturer’s protocols, we extracted total RNA from tissues or cells using Ultrapure RNA Kit (CoWin Biosciences, China). Then the concentration was measured by a multi-wavelength microplate reader Tecan’s Infinite M200 Pro (Thermo Fisher Scientific, USA). Afterwards, PrimeScript™ RT Master Mix (Takara, Japan) was applied to transform the RNA solution into cDNA solution. The qPCR conditions were seen in the manufacturer’s protocols. GAPDH was considered as an endogenous control. All qRT-PCR assays in the paper were conducted in triplicate. TSINGKE provided us with the forward or reverse primers for CENPA and GAPDH. The primer sequences used for qPCR were: CENPA: 5′-GTG TGG ACT TCA ATT GGC AAG-3′ (forward) and 5′-TGC ACA TCC TTT GGG AAG AG-3′(reverse); CTNNB1: 5′-AAA GCG GCT GTT AGT CAC TGG-3′ (forward) and 5′-CGA GTC ATT GCA TAC TGT CCA T-3′(reverse); GAPDH: 5′-CGT GGA AGG ACT CAT GAC CA-3′ (forward) and 5′-GCC ATC ACG CCA CAG TTT C-3′ (reverse).

### IHC assay

Briefly, immunohistochemical was stained with 4 µm formalin‐fixed paraffin‐embedded tissue sections. The slices were then reacted with a rabbit antibody against CENPA (1:100) for 12 h at 4 °C. Then the section was washed with PBS, immunodetection was performed with 50 µl DAKO secondary antibody per section and cultured with secondary antibodies at 25 °C for about 2 h. Three randomly fields were selected to observe under a light microscope (Olympus in Japan) at 200× and 400× magnification.

### Transient transfection for overexpression or knockdown of CENPA and/or CTNNB1

Plasmids overexpressing CENPA, siRNA targeting CENPA (si-CENPA) oligonucleotide sequences and their corresponding negative controls were constructed in Vigene Biosciences (Shandong, China). The siRNA targeting CTNNB1 and the corresponding negative control siRNA were synthesized by Wuhan Qijing Biological Technology (Wuhan, China). Lipofectamine 3000 (Invitrogen, Carlsbad, CA) reagent was employed for transfection directed by the manufacturer’s protocols while the ccRCC cells were at 30–50% fusion. 5 μg per well of plasmids (vector or CENPA) or 0.1 nmol per well of siRNAs (si-CENPA, si-CTNNB1 or si-NC) were used directed by the manufacturer's protocols. Finally, cells were stored for further experiments after 48 h transient transfection. Si-CENPA sequence was as follows: sense 5′-GCA GCA GAA GCA UUU CUA GUU TT-3′; antisense 5′-AAC UAG AAA UGC UUC UGC UGC TT-3′. The si-CTNNB1 sequence was as follows: sense 5′-GGA UGU GGA UAC CUC CCA ATT-3′; antisense 5′-UUG GGA GGU AUC CAC AUC CTC-3′.

### Cell proliferation assays and cell cycle analysis

After transient transfection for at least 48 h, 1 × 10^3^ cells were cultured in 96-well plate with 200 µl of medium. The cell proliferation assays were measured by CCK-8 (MedChemExpress, USA) at a 1:10 dilution with serum-free medium every 24 h for four days directed by the manufacturer’s protocols. Finally, OD_450_ of cells over four days was shown by GraphPad Prism to reflect the ability of cell proliferation. As for cell cycle analysis, Caki-1 cells were labeled with PI/RNase Staining (BD Bioscience) Buffer after transfected with Si-CENPA for 48 h. The DNA content was measured using flow (Beckman FC500, USA) cytometry and displayed by Modfit software.

### Cell migration and invasion assays

The standard steps are as described in the previous [64]. Notably serum-starved cells (A498: 2 × 10^4^; Caki-1: 10^5^) were used for migration assays. For invasion assays, the cells were double.

### Bioinformatics analyses

The ccRCC samples were classified into two groups with the same sample based on the median CENPA expression. The GSEA (http://www.broadinstitute.org/gsea) analysis was conducted for enrichment analysis according to the grouping. The p < 0.05 and the false discovery rate (FDR) value < 0.25 were thoughted as the relevant enriched pathways [65]. The KEGG and GO analyses of differential expressed genes form two CENPA expression groups were conducted by R 4.0.2.

### The activation or inhibition of Wnt signaling pathway

10 μmol/L Wnt/β-catenin inhibitor XAV-939 or agonist CHIR-99021 trihydrochloride (MedChemExpress, USA, HY-15147 and HY-10182B) were mixed in cells in 6-well plates for 24 h when confluence reached 60%. Then the proteins were collected for further analysis.

### Wound healing assay

The same amounts of cells were cared in the 6-well plates. When the fusion reached 100%, cells were wounded with the same size. Pictures of wounds were observed at 24 h.

### Colony formation assays

1000 A498 (Si-NC, Si-CENPA, Vector, CENPA) and Caki-1 (Si-NC, Si-CENPA, Vector, CENPA) cells were cultured into a well of 6-well plates. After about 12 days, the cells were fixed for 30 min. After washed by PBS the cells were then dyed with crystal violet for 40 min.

### Statistical analyses

The group data were presented with mean and standard deviation (SD). The differences between groups were evaluated using a Student's test or paired Student's test. The relationships between CENPA expression and clinicopathological characteristics of ccRCC samples were analyzed by Pearson's χ2 test. To conduct univariate and multivariate Cox regression analyses, we assigned clinicopathological features as binary variables, including CENPA mRNA expression levels and set OS as dependent variable. Then the analysis were conducted by SPSS 25.0. Significance was determined at *P* < 0.05. All of experiments were repeated for three times. Except the qRT-PCR data of tumor and normal tissues were represented as mean, the rest were represented as the mean ± SD. All analyses above were conducted by GraphPad Prism (GraphPad Software, San Diego, California in USA) as seen in previous article [66].

## Supplementary Information

**Additional file 1: Figure S1.** CENPA was closely related to clinical traits. (A-B) The expression of CENPA elevated with N stage and M stage in TCGA-KIRC cohort. (C) The ROC curve of CENPA in TCGA KIRC N/T pairs. (D-G) The subgroup analysis of survival curve according to age, gender, Stage and G grade and the results were similar to the previous. (H–K) The subgroup analysis of ROC curve according to T, M, Stage and G grade. (L–M) CENPA expression of KIRC patients without adjuvant therapy prior to the surgery.

**Additional file 2: Figure S2.** The GO and KEGG analysis of CENPA. (A-B) The results of the GO and KEGG analysis about CENPA. TCGA-KIRC ccRCC samples were divided into two groups based on CENPA expression levels, then DEGs between two groups were enriched referred to GO and KEGG database. (C, E, F and H) To explore their effects on β-catenin (CTNNB1), a series of concentration gradients for 24 h and time gradients for 10 μM of XAV-939 and CHIR-99021 trihydrochloride were employed Caki-1. Then, 10 uM and 24 h were considered to be a proper drug treatment condition. (D and G) The mRNA and protein expression of CTNNB1 in A498 and Caki-1 cells after transfected with si-CTNNB1. (I) The genetic alteration information of eight CNEP family members.

**Additional file 3: Table S1.** The clinicopathological features of the 120 ccRCC patients.

**Additional file 4.** The original western blot pictures.

## Data Availability

The datasets and data used in this study can be obtained from official website or corresponding author.

## Abbreviations

RCC: Renal cell carcinoma
ccRCC: Clear cell renal cell carcinoma
DEG: Differentially expressed genes
CENP: Centromere protein
WGCNA: Weighted gene co-expression network analysis
KIRC: Kidney renal clear cell carcinoma
TCGA: The Cancer Genome Atlas
CNA: Copy-number alteration
OS: Overall survival
DFS: Disease free survival
CCLE: Cancer Cell Line Encyclopedia
GEO: Gene expression omnibus
ROC: Receiver operator characteristic
qRT-PCR: Reverse transcription-quantitative PCR
IHC: Immunohistochemistry
IBT: Immunoblotting test
CCK-8: Cell Counting Kit-8
GSEA: Gene set enrichment analysis
GO: Gene Ontology
KEGG: Kyoto Encyclopedia of Genes and Genomes
CTNNB1: β-Catenin
CCND1: Cyclin D1
DEG: Differential expressed gene
RNA-seq: RNA sequencing
AJCC: American Joint Committee on Cancer
PBST: Phosphate Buffered Saline Tween
FDR: False discovery rate
SD: Standard Deviation
