# Supplementary figures and images for: CENPA promotes clear cell renal cell carcinoma progression and metastasis via Wnt/β-catenin signaling pathway
Source: J Transl Med. 2021 Oct 9;19:417. doi: 10.1186/s12967-021-03087-8 (PMC8502268; doi:10.1186/s12967-021-03087-8)

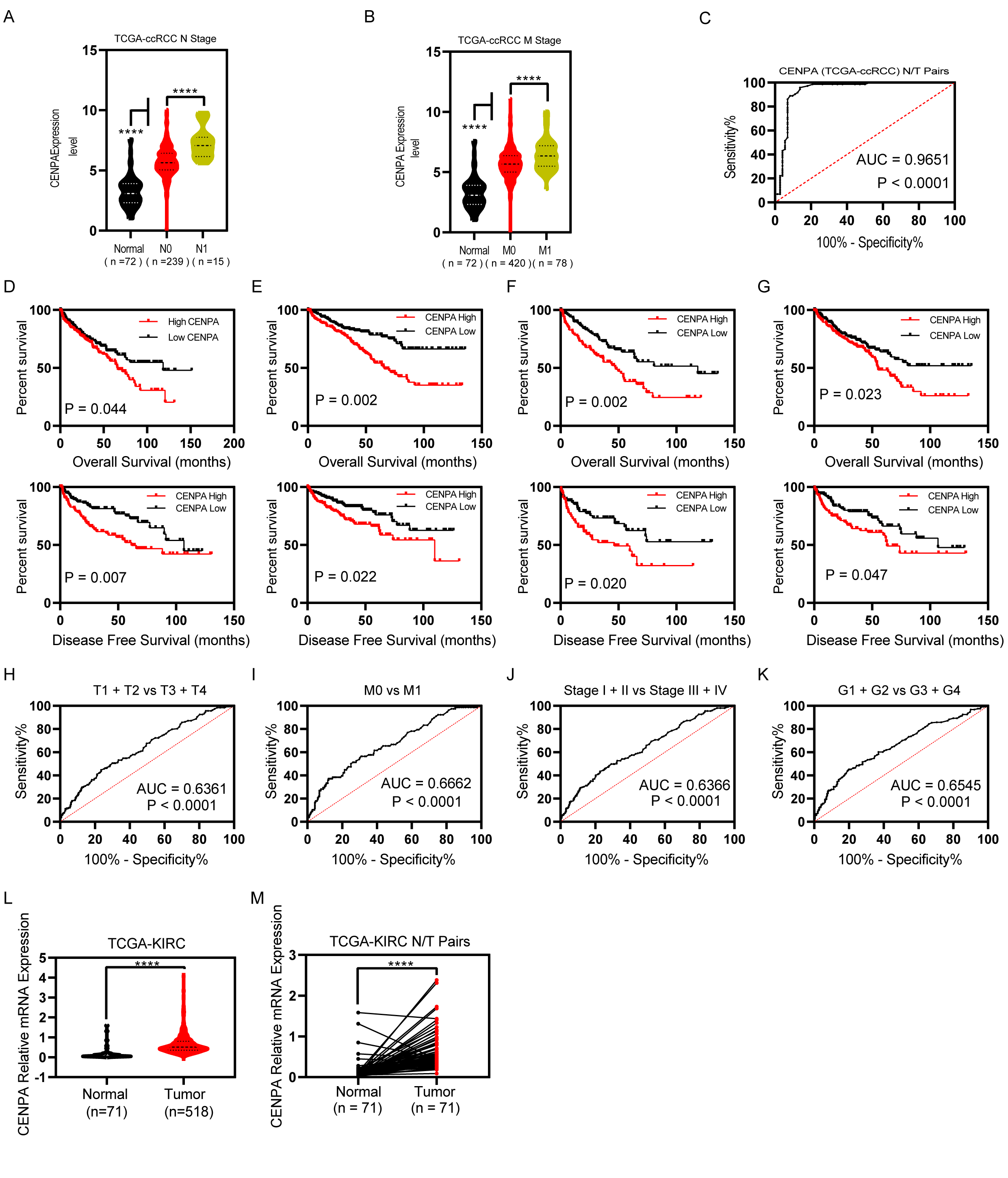

Supplement: Supplementary file 1 — Additional file 1: Figure S1. CENPA was closely related to clinical traits. (A-B) The expression of CENPA elevated with N stage and M stage in TCGA-KIRC cohort. (C) The ROC curve of CENPA in TCGA KIRC N/T pairs. (D-G) The subgroup analysis of survival curve according to age, gender, Stage and G grade and the results were similar to the previous. (H–K) The subgroup analysis of ROC curve according to T, M, Stage and G grade. (L–M) CENPA expression of KIRC patients without adjuvant therapy prior to the surgery. [file 12967_2021_3087_MOESM1_ESM.tif]

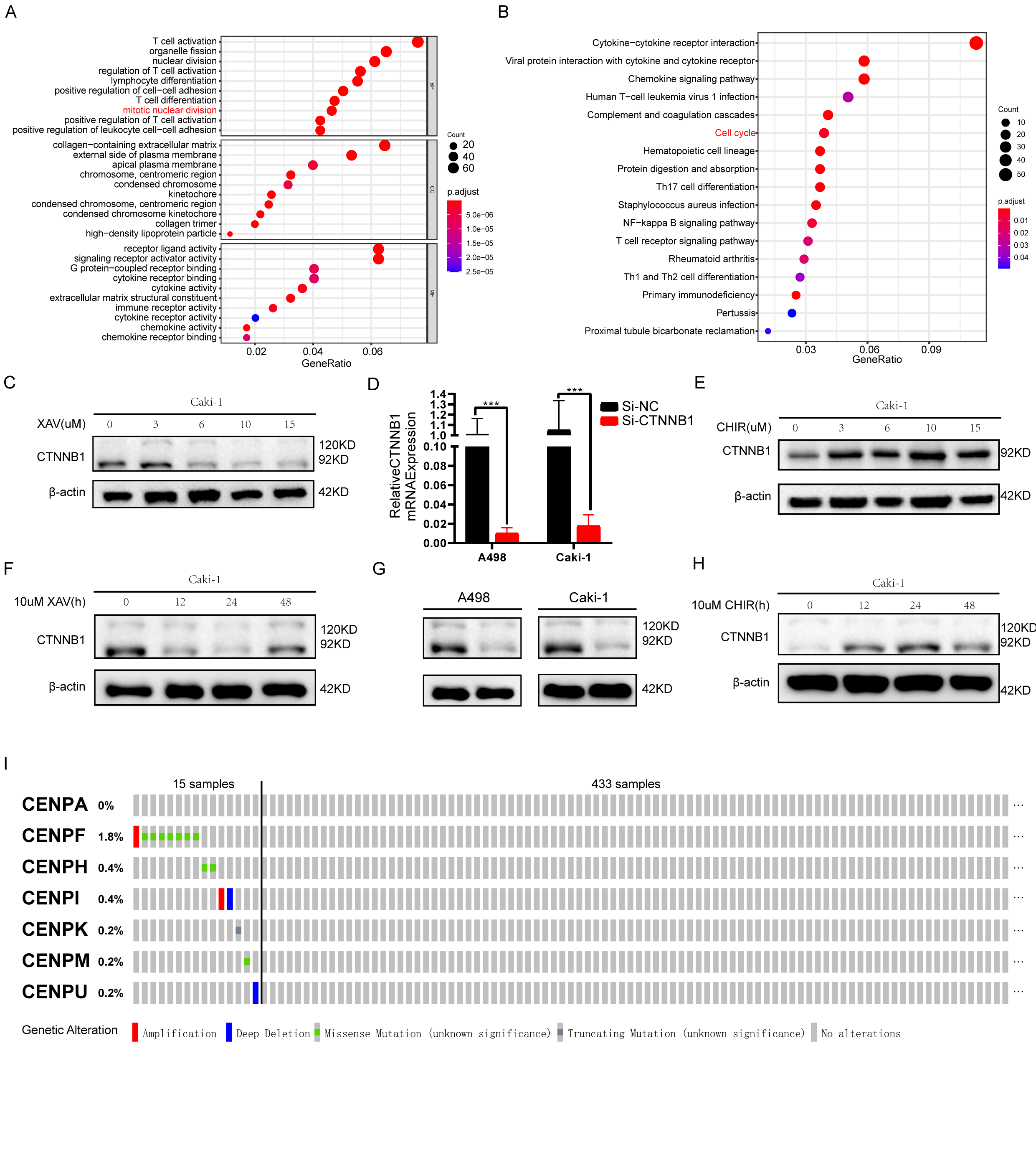

Supplement: Supplementary file 2 — Additional file 2: Figure S2. The GO and KEGG analysis of CENPA. (A-B) The results of the GO and KEGG analysis about CENPA. TCGA-KIRC ccRCC samples were divided into two groups based on CENPA expression levels, then DEGs between two groups were enriched referred to GO and KEGG database. (C, E, F and H) To explore their effects on β-catenin (CTNNB1), a series of concentration gradients for 24 h and time gradients for 10 μM of XAV-939 and CHIR-99021 trihydrochloride were employed Caki-1. Then, 10 uM and 24 h were considered to be a proper drug treatment condition. (D and G) The mRNA and protein expression of CTNNB1 in A498 and Caki-1 cells after transfected with si-CTNNB1. (I) The genetic alteration information of eight CNEP family members. [file 12967_2021_3087_MOESM2_ESM.tif]
