# Supplementary material for: CENPA promotes clear cell renal cell carcinoma progression and metastasis via Wnt/β-catenin signaling pathway
Source: J Transl Med. 2021 Oct 9;19:417. doi: 10.1186/s12967-021-03087-8 (PMC8502268; doi:10.1186/s12967-021-03087-8)
Supplement: Supplementary file 4 — Additional file 4. The original western blot pictures. [file 12967_2021_3087_MOESM4_ESM.pptx]

## Slide 1
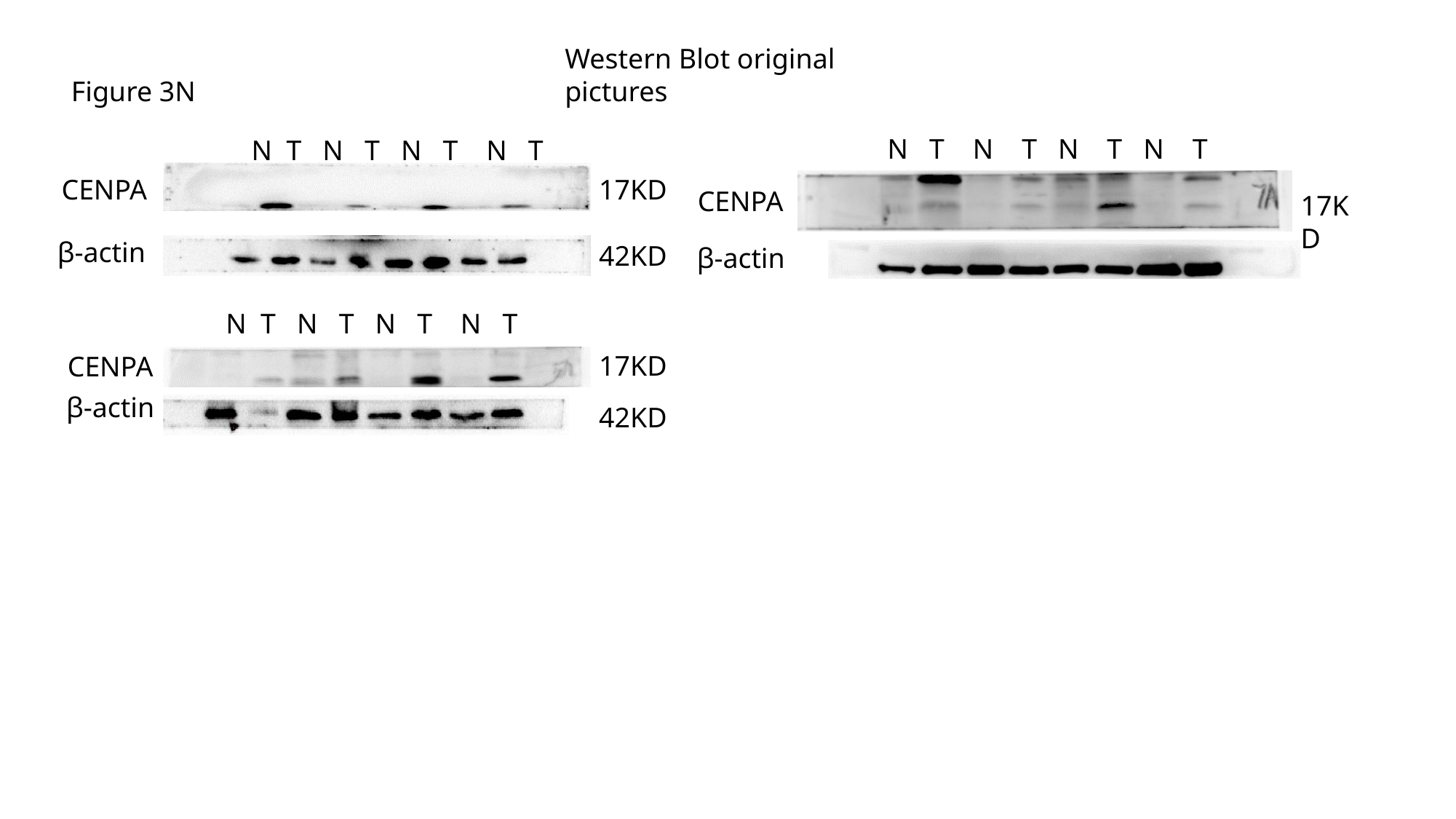

Western Blot original pictures
Figure 3N
N T N T N T N T
N T N T N T N T
CENPA
17KD
CENPA
17KD
β-actin
42KD
β-actin
N T N T N T N T
17KD
CENPA
β-actin
42KD

## Slide 2
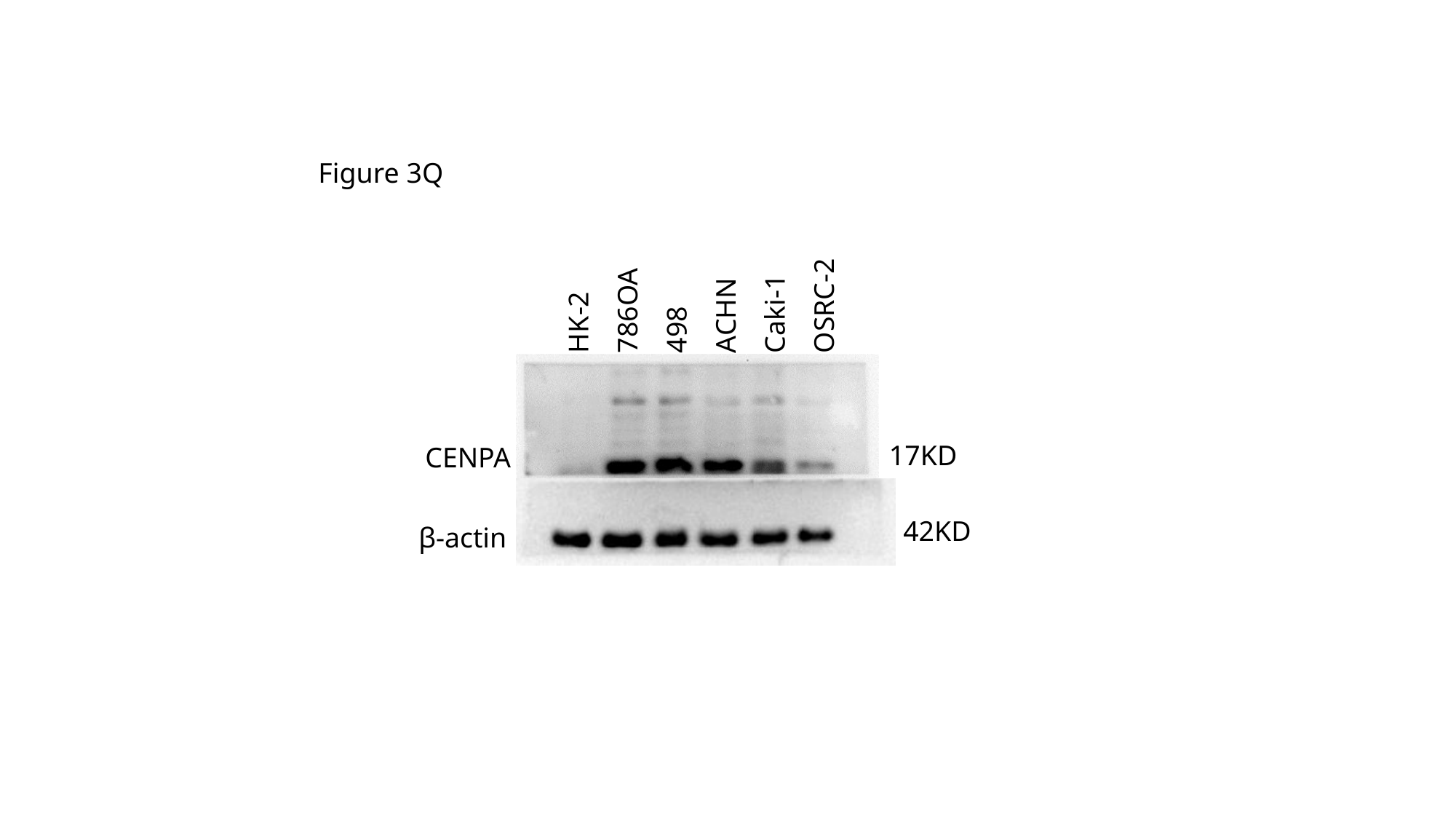

Figure 3Q
HK-2
786OA498
ACHN
Caki-1
OSRC-2
17KD
CENPA
42KD
β-actin

## Slide 3
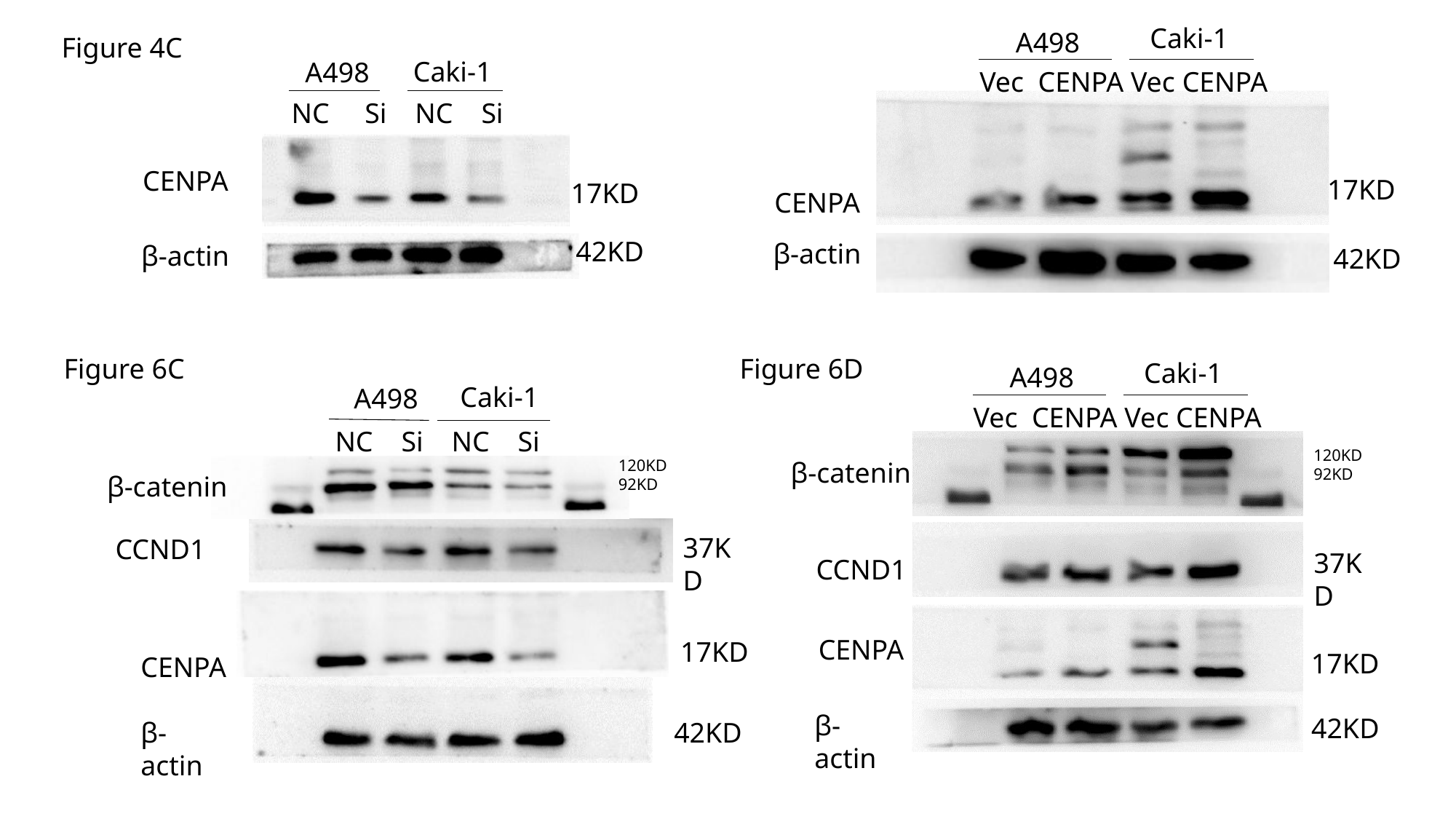

Caki-1
A498
Figure 4C
Caki-1
A498
Vec CENPA Vec CENPA
NC Si NC Si
CENPA
17KD
17KD
CENPA
42KD
β-actin
β-actin
42KD
Figure 6C
Figure 6D
Caki-1
A498
Caki-1
A498
Vec CENPA Vec CENPA
NC Si NC Si
120KD
92KD
120KD
92KD
β-catenin
β-catenin
37KD
CCND1
37KD
CCND1
CENPA
17KD
17KD
CENPA
β-actin
42KD
β-actin
42KD

## Slide 4
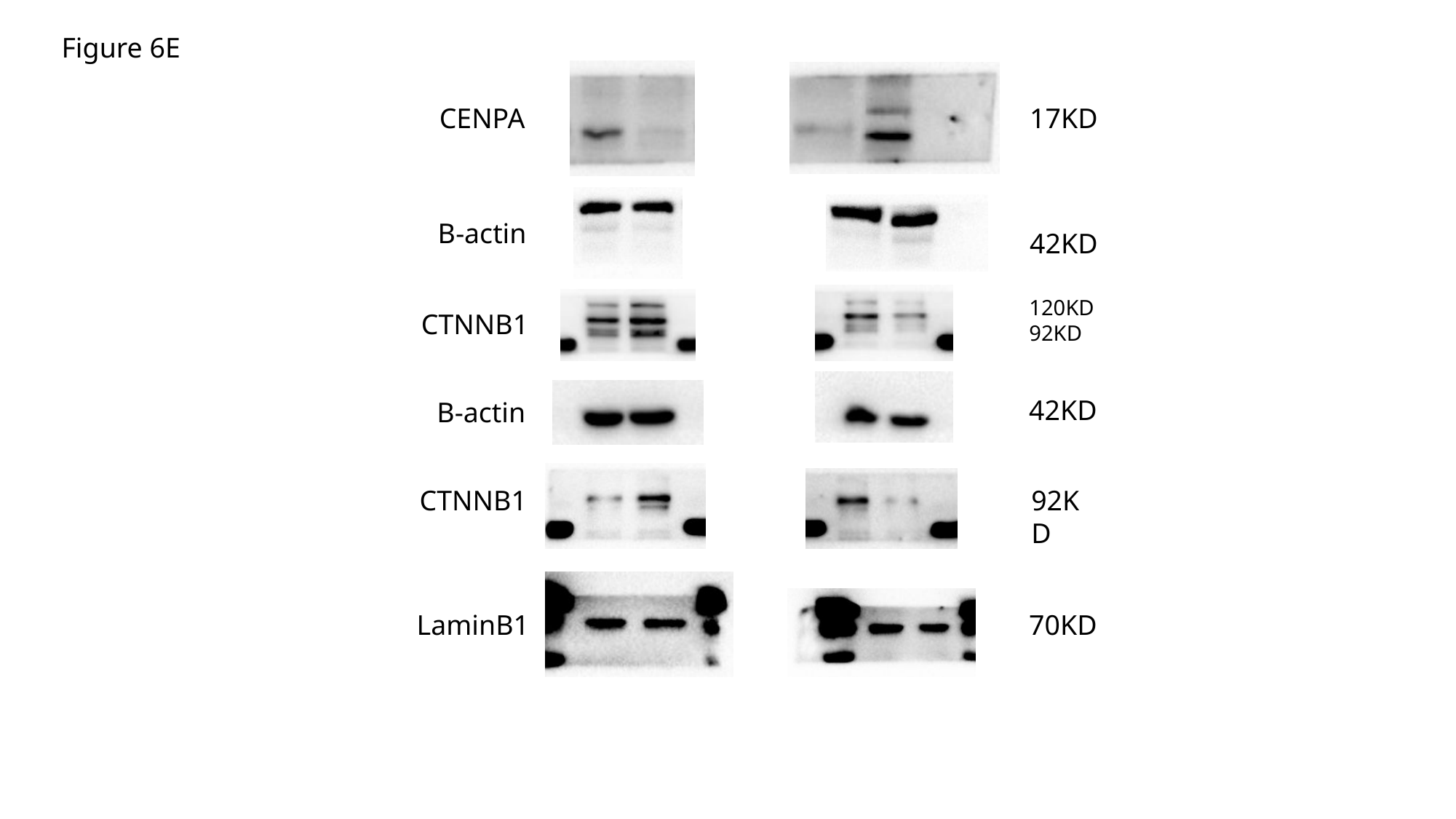

Figure 6E
17KD
CENPA
Β-actin
42KD
120KD
92KD
CTNNB1
42KD
Β-actin
CTNNB1
92KD
LaminB1
70KD

## Slide 5
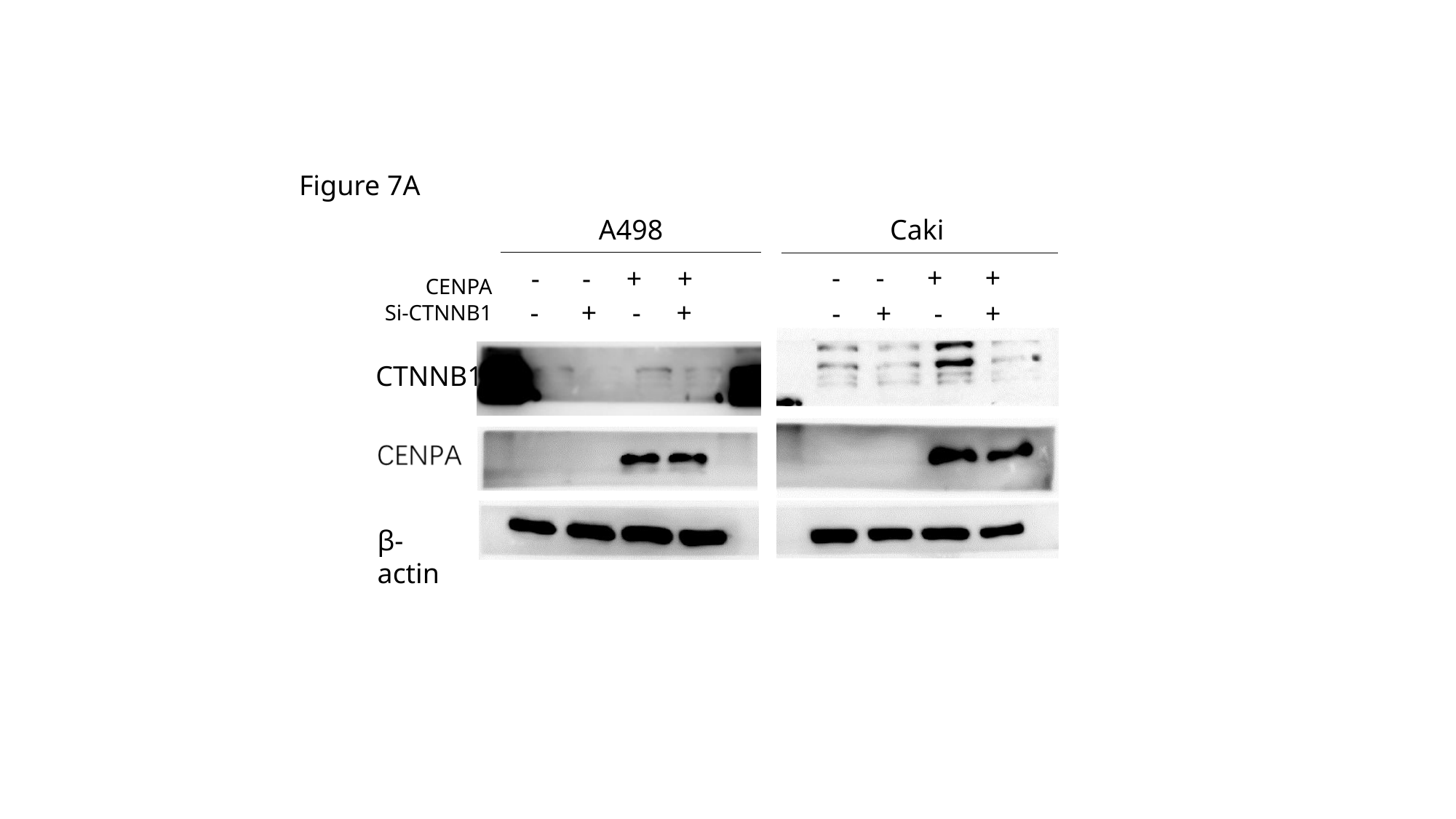

Figure 7A
A498
Caki
- - + +
- - + +
CENPA
Si-CTNNB1
- + - +
- + - +
CTNNB1
β-actin

## Slide 6
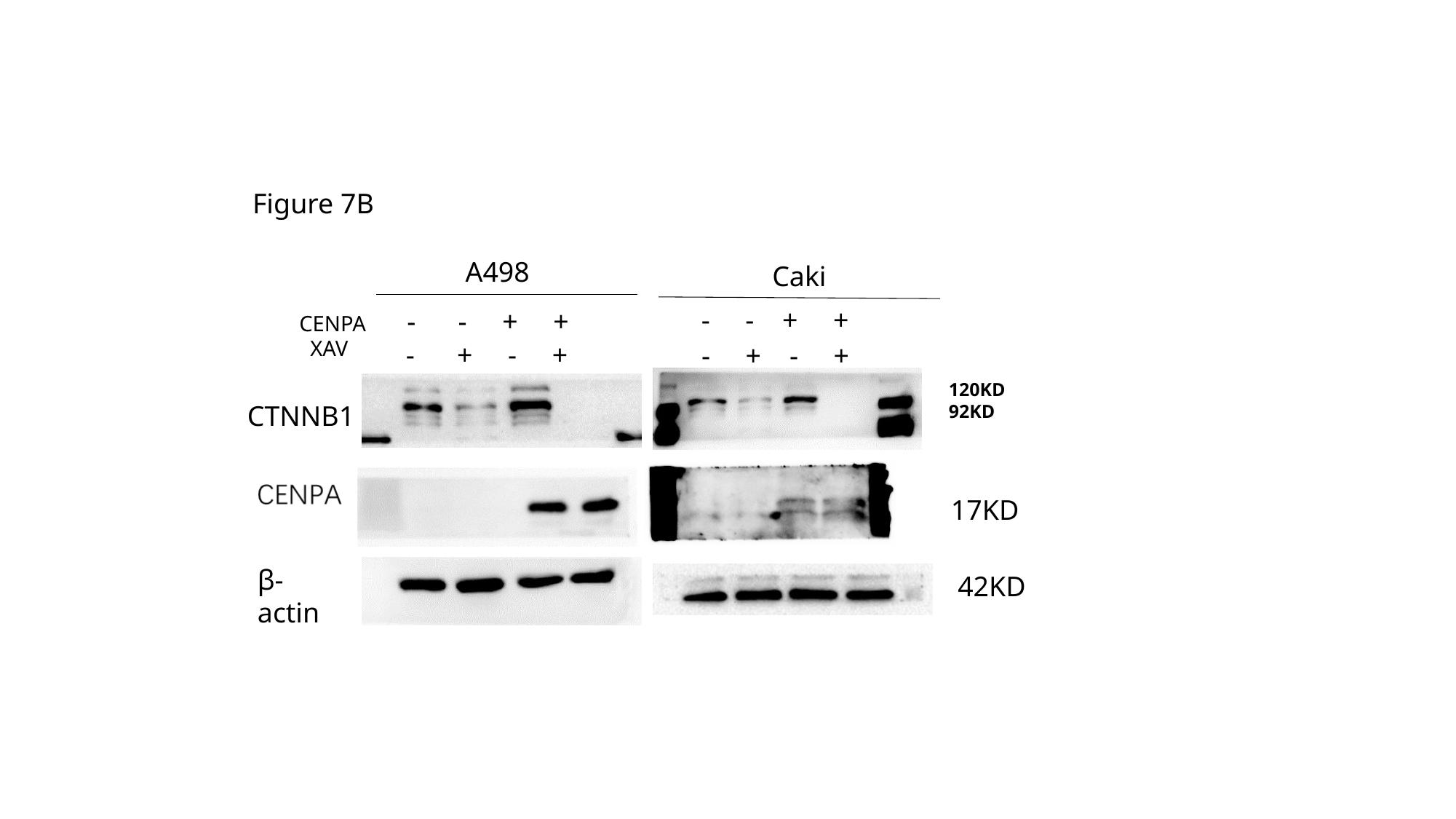

Figure 7B
A498
Caki
- - + +
- - + +
CENPA
 XAV
- + - +
- + - +
120KD
92KD
CTNNB1
17KD
β-actin
42KD

## Slide 7
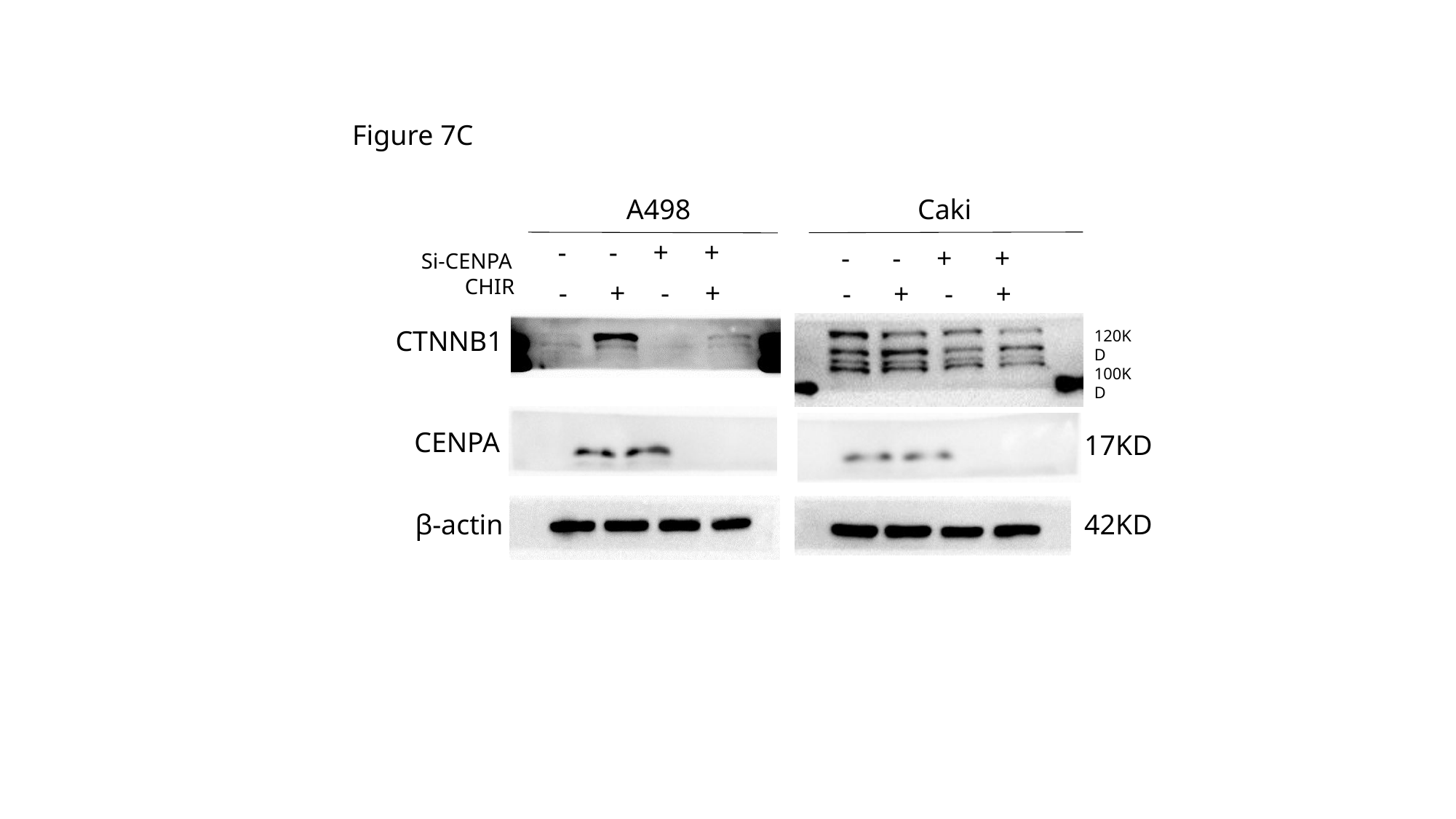

Figure 7C
A498
Caki
- - + +
- - + +
Si-CENPA
 CHIR
- + - +
- + - +
CTNNB1
120KD
100KD
CENPA
17KD
β-actin
42KD

## Slide 8
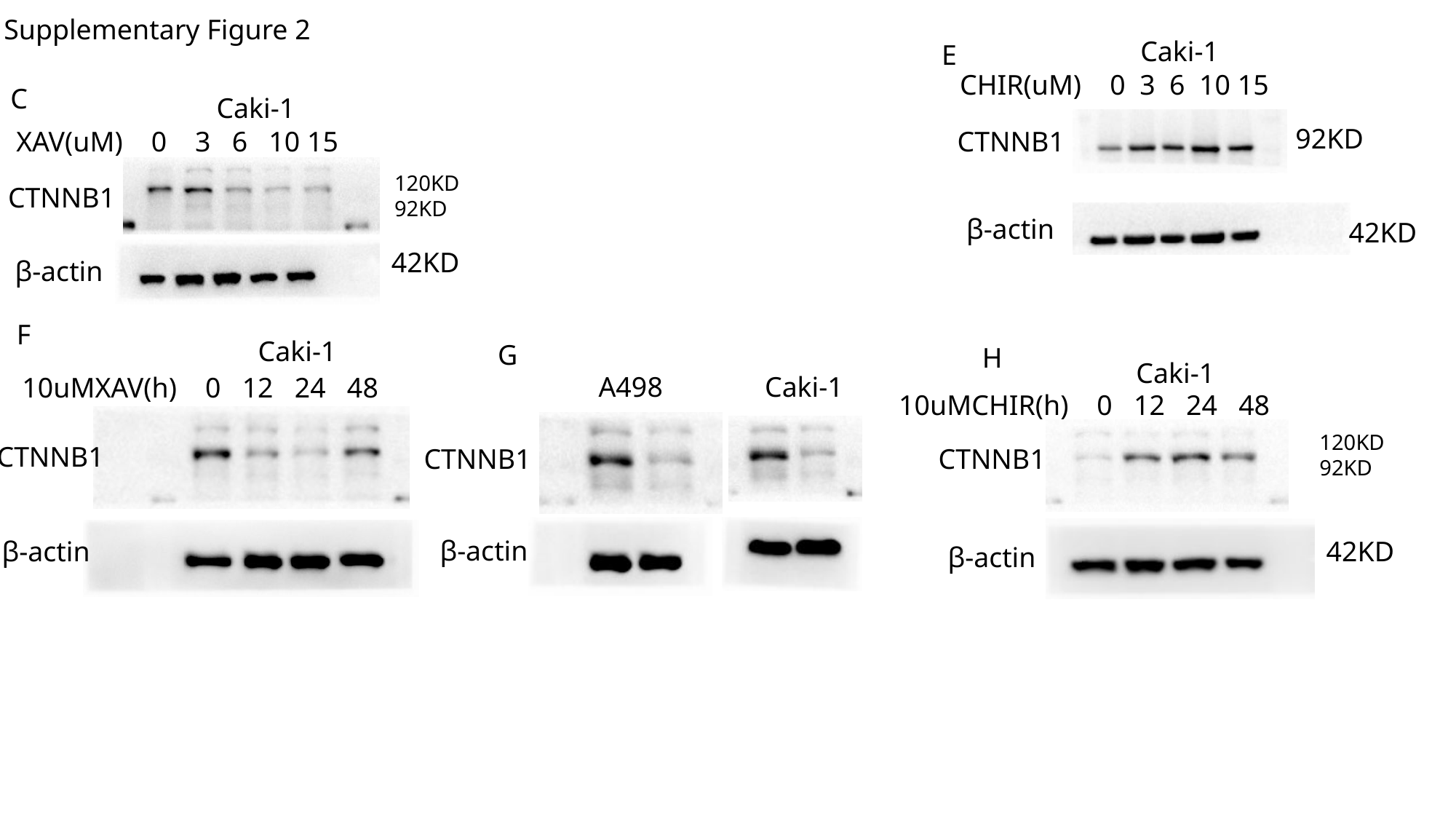

Supplementary Figure 2
Caki-1
E
CHIR(uM) 0 3 6 10 15
C
Caki-1
92KD
XAV(uM) 0 3 6 10 15
CTNNB1
120KD
92KD
CTNNB1
β-actin
42KD
42KD
β-actin
F
Caki-1
G
H
Caki-1
A498
Caki-1
10uMXAV(h) 0 12 24 48
10uMCHIR(h) 0 12 24 48
120KD
92KD
CTNNB1
CTNNB1
CTNNB1
β-actin
β-actin
42KD
β-actin
